# Development of a novel HDAC6 PET imaging agent uncovers associations between HDAC6 overexpression and neuroinflammation in depression

**DOI:** 10.1016/j.redox.2026.104014

**Published:** 2026-01-12

**Authors:** Yanting Zhou, Yuheng Zou, Xiao Zhong, Hongyan Li, Jingyi Yang, Hui Meng, Weiyao Xie, Pan Yao, Xiaoai Wu, Huawei Cai, Lin Li, Changning Wang, Wei Zhang, Ping Bai

**Affiliations:** a Department of Pulmonary and Critical Care Medicine, West China Hospital, State Key Laboratory of Respiratory Health and Multimorbidity, Sichuan University, Chengdu, Sichuan, 610041, China; b Molecularly Targeted Research and Development Laboratory, Institute of Respiratory Health, Frontiers Science Center for Disease-related Molecular Network, West China Hospital, Sichuan University, Chengdu, Sichuan, 610041, China; c Precision Medicine Center, Precision Medicine Key Laboratory of Sichuan Province, West China Hospital, Sichuan University, Chengdu, Sichuan, 610041, China; d The Research Units of West China, Chinese Academy of Medical Sciences, West China Hospital, Chengdu, Sichuan, 610041, China; e Institute of Respiratory Health and Multimorbidity, West China Hospital, Sichuan University, Chengdu, Sichuan, 610041, China; f Department of Nuclear Medicine, Laboratory of Clinical Nuclear Medicine, West China Hospital, Sichuan University, Chengdu, Sichuan, 610041, China; g West China Biomedical Big Data Center, West China Hospital, Sichuan University, Chengdu, Sichuan, 610041, China; h Mental Health Center and Psychiatric Laboratory, The State Key Laboratory of Biotherapy, West China Hospital, Sichuan University, Chengdu, Sichuan, 610041, China; i Athinoula A. Martinos Center for Biomedical Imaging, Department of Radiology, Massachusetts General Hospital, Harvard Medical School, Charlestown, MA, 02129, United States

**Keywords:** HDAC6, PET imaging, Radiotracer, Major depressive disorder, Neuroinflammation

## Abstract

Histone deacetylase 6 (HDAC6) represents a compelling target in major depressive disorder (MDD) pathophysiology, yet *in vivo* investigation has been constrained by inadequate imaging capabilities. Here, we report the development and validation of [^18^F]PB200, a novel positron emission tomography (PET) radiotracer specifically targeting brain HDAC6. PB200 was engineered with nanomolar affinity, high HDAC6 selectivity, and excellent blood-brain barrier permeability. [^18^F]PB200 was successfully synthesized in a radiochemical yield of 13 ± 4 % and validated through *in vitro* autoradiography and *in vivo* PET imaging across rodent and non-human primate models. We subsequently employed [^18^F]PB200 alongside TSPO-targeted [^18^F]FEPPA PET imaging in a chronic unpredictable mild stress (CUMS) mouse model of depression. This dual-tracer approach, complemented by *in vitro* experiments, revealed significant HDAC6 upregulation occurring concurrently with enhanced neuroinflammatory markers, including microglial activation and elevated pro-inflammatory cytokines. Our findings provide the first *in vivo* molecular imaging evidence directly linking HDAC6 upregulation to depressive pathophysiology and associated neuroinflammation. This work illuminates the molecular relationship between depression and neuroinflammation while establishing [^18^F]PB200 as a valuable tool for evaluating HDAC6-targeted therapeutic interventions, potentially advancing precision diagnosis and treatment approaches for depression.

## Graphical abstract

**Figure:**
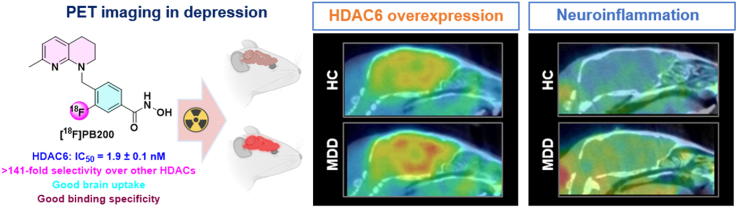

## 1 Introduction

Major Depressive Disorder (MDD) represents a significant global health burden, affecting over 350 million people worldwide with high morbidity, disability, and mortality rates [1,2]. The complex and incompletely understood pathophysiology of depression, coupled with a lack of specific symptoms and objective diagnostic criteria, has resulted in limited therapeutic efficacy [3,4]. First-line pharmacological interventions, including selective serotonin reuptake inhibitors (SSRIs) and serotonin-norepinephrine reuptake inhibitors (SNRIs), primarily target neurotransmitter dysfunction; however, approximately two-thirds of patients fail to achieve remission following initial treatment, with one-third remaining treatment-resistant despite multiple therapeutic approaches [5,6]. The multifaceted pathology of MDD extends beyond neurotransmitter imbalances to encompass genetic factors, epigenetic modifications, neuroinflammation, impaired neuroplasticity, mitochondrial dysfunction, oxidative stress, and gut microbiome alterations [7,8]. This complexity explains the limitations of current pharmacological strategies and underscores the critical need for further research into novel diagnostic and therapeutic targets.

Recent investigations have revealed compelling associations between the epigenetic regulator histone deacetylase 6 (HDAC6) and MDD [[9], [10], [11]]. HDAC6, a class IIb deacetylase predominantly localized in the cytoplasm, possesses unique dual catalytic domains and a ubiquitin-binding domain that interacts with various proteins [12]. Its canonical function involves deacetylation of α-tubulin, thereby regulating microtubule dynamics and axonal transport. HDAC6 also participates in stress granule formation, autophagy regulation, protein degradation, and various signaling pathways, making it intricately linked to cellular stress responses, inflammation, and neurological pathologies [13,14]. Studies show that the expression of HDAC6 is significantly elevated in specific brain regions MDD compared to controls. This finding is consistent with microtubule instability and impaired neuroplasticity [9,15]. Preclinical research has demonstrated that selective HDAC6 inhibition, through compounds such as ACY-738 and ACY-775, produces antidepressant-like effects by enhancing synaptic plasticity and modulating stress response mechanisms [16,17]. The antidepressant action of HDAC6 inhibitors potentially involves suppression of neuroinflammatory pathways activated in depression, reducing the release of inflammatory cytokines including IL-1β, IL-6, and TNF-α. These findings suggest HDAC6 as a promising diagnostic and therapeutic target for MDD, though its precise pathological role requires further elucidation [18,19].

Positron emission tomography (PET) imaging technology offers unprecedented capabilities for non-invasive visualization and quantification of molecular targets *in vivo* [20,21]. The development of HDAC6-specific PET imaging probes could potentially bridge the gap between preclinical observations and clinical applications by enabling direct assessment of HDAC6 expression and function in depression. Such molecular imaging tools would not only advance our understanding of HDAC6's role in MDD pathophysiology but also facilitate the evaluation of HDAC6-targeted therapeutic interventions through measurement of target engagement. While the development of HDAC6 radioligands has advanced, substantial obstacles persist in translating these compounds into clinically useful imaging agents [[22], [23], [24], [25], [26]]. The current research landscape in HDAC inhibitor development is characterized by a concerted focus on creating highly specific, low-nanomolar potency HDAC6 inhibitors; however, optimizing brain distribution and kinetic properties of these molecules remains a significant challenge. A predominant limitation of hydroxamic acid-based HDAC6 radioligands is their inadequate blood-brain barrier (BBB) permeability, attributable to excessive polarity and insufficient metabolic stability. Our previous investigations led to the development of [^11^C]Martinostat, which demonstrated favorable brain uptake and has undergone multiple human studies, yet its binding affinity extends to HDAC1, 2, 3, and 6, exhibiting suboptimal selectivity for HDAC6 [[27], [28], [29]]. Subsequently, [^18^F]Bavarostat emerged as the first brain-penetrant HDAC6 PET probe with successful translation to human studies. Nevertheless, its widespread application is constrained by complex radiosynthetic requirements, including multiple reaction steps and specialized ruthenium complexes [23,30]. Additionally, we previously developed [^18^F]PB118, which exhibits commendable brain uptake and *in vivo* binding specificity, with investigations extending to Alzheimer's disease pathology; however, this radioligand has not yet progressed to clinical evaluation.

In the present study, we detail our comprehensive strategy for developing a novel HDAC6 PET probe with improved binding specificity, enhanced BBB penetration, and improved metabolic stability based on our previously discovered HDAC6 inhibitor PB131 [31]. Through systematic structural optimization and evaluation, we identified inhibitor PB200 as a promising candidate for HDAC6 PET imaging probe development. We successfully radiolabeled PB200 with fluorine-18 and validated [^18^F]PB200 through comprehensive *in vitro* and *in vivo* experiments, confirming its efficacy as a high-performance HDAC6 PET radiotracer. Furthermore, we utilized this newly developed radioligand to investigate HDAC6's role in the pathophysiology of depression, providing valuable insights into potential therapeutic targets and innovative strategies for both the diagnosis and treatment of MDD.

## 2 Results

### 2.1 Synthesis and biological evaluation of HDAC6 inhibitors as PET probe candidates

**Fig. 1:**
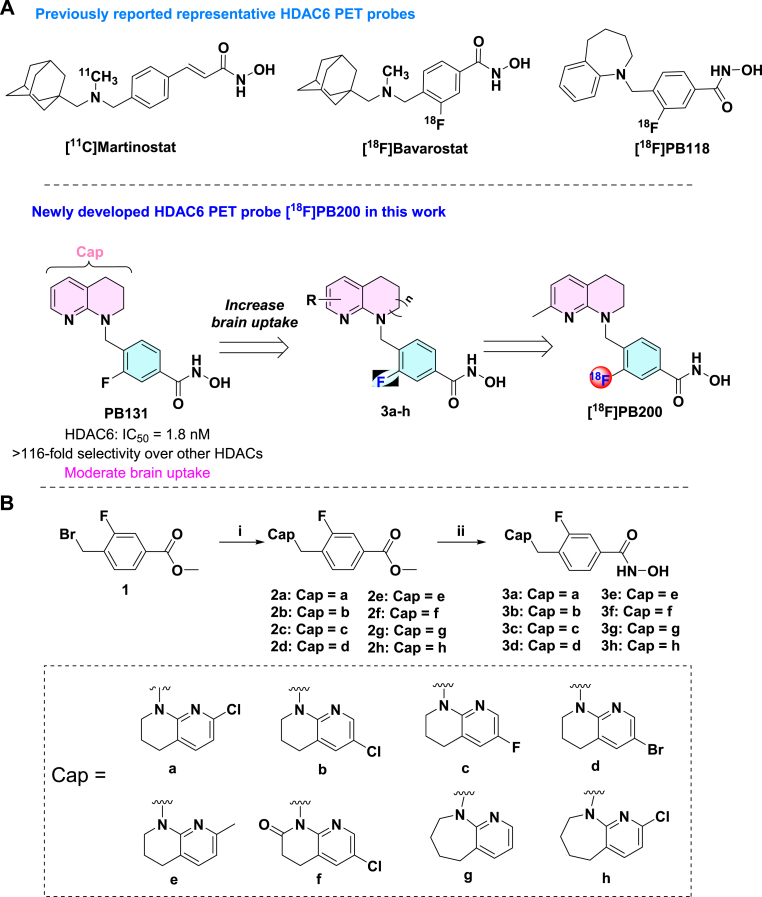
Design and synthesis of HDAC6 inhibitors. (A) Representative HDAC6 PET probes reported previously ([^11^C]Martinostat, [^18^F]Bavarostat, and [^18^F]PB118) and the optimization of PB131 for developing [^18^F]PB200. (B) Synthesis of compounds **3a**-**3h**. Reaction conditions: (i) **2a-g**: corresponding amine derivatives, NaHMDS, THF, −78 °C, 30 min; **2h**: 7-chloro-3,4-dihydro-1H-1,8-naphthyridin-2-one, NaH, DMF, 0∼r.t; (ii) NH_2_OH·HCl, LiOH, MeOH, r. t.

We conducted strategic structural optimization of the lead compound PB131, focusing primarily on modifications to the Cap region. Our design strategy aimed to enhance brain uptake while preserving the compound's high affinity and selectivity for HDAC6 (Fig. 1A). As illustrated in Fig. 1B, we successfully synthesized a series of eight novel inhibitors (**3a-h**) through a concise two-step synthetic route involving nucleophilic substitution followed by hydroxamic acid formation.

Enzymatic assays revealed that all synthesized compounds exhibited remarkable potency against HDAC6, with IC_50_ values ranging from 1.97 to 8.11 nM (Table 1). Of note, compound **3e** demonstrated exceptional inhibitory activity with the lowest IC_50_ value of 1.97 nM. Structure-activity relationship (SAR) analysis demonstrated that the positioning of halogen substituents proved critical, with compound **3a** (chlorine at 2-position) exhibiting greater potency than its regioisomer **3b** (chlorine at 1-position). Moreover, the fluorine-substituted analog **3c** (IC_50_ = 2.62 ± 0.11 nM) demonstrated superior activity compared to both chloro and bromo derivatives, suggesting that smaller, more electronegative halogens confer optimal interactions with the binding pocket.

**Table 1:** The enzymatic activity and physicochemical property of synthetic compounds.^a^.

Cpd.	Cap	HDAC6(IC_50_, nM)	CNS parameters			
			MW	tPSA	cLogD	CNS MPO
3a		6.01 ± 0.21	336.1	64.9	3.0	5.3
3 b		4.15 ± 0.26	336.1	64.9	3.0	5.3
3c		2.62 ± 0.11	320.1	64.9	2.5	5.6
3 d		8.11 ± 0.34	380.0	64.9	2.9	5.2
3e		1.97 ± 0.18	316.1	64.9	2.8	5.4
3f		5.67 ± 0.37	350.1	64.9	3.2	5.1
3 g		3.89 ± 0.30	316.1	64.9	2.8	5.43
3 h		2.74 ± 0.19	350.1	82.2	1.7	5.7
TSA	–	2.07 ± 0.24	–	–	–	

a IC_50_ values were determined against HDAC6 using a 10-point, 3-fold serial dilution starting at 1 μM. The results are expressed as the mean ± SD, with Trichostatin A (TSA) serving as the positive control. Each experiment was repeated three times. cLog*P* and tPSA are calculated by ChemBioDraw Ultra 16.0.

To evaluate the potential of these compounds as neuroimaging agents, we conducted a comprehensive assessment of their physicochemical properties, specifically calculating the central nervous system multi-parameter optimization (CNS MPO) scores based on molecular weight (MW), tPSA, and cLogD. These parameters serve as critical predictors of BBB permeability and pharmacokinetic suitability. As detailed in Table 1, all eight compounds displayed highly favorable profiles with CNS MPO scores ranging from 5.1 to 5.7, significantly surpassing the standard threshold (score ≥4.0) for CNS candidates. The series exhibited optimal lipophilicity (cLogD: 1.7–3.2) and appropriate polarity (tPSA: 64.9–82.2 Å). Notably, compound **3e** (renamed PB200) was identified as the lead candidate; it combined the highest inhibitory potency (IC_50_​ = 1.97 nM) with a desirable physicochemical profile (CNS MPO = 5.4), warranting its further development as a PET neuroimaging probe.

### 2.2 Target binding selectivity assessments of PB200

**Fig. 2:**
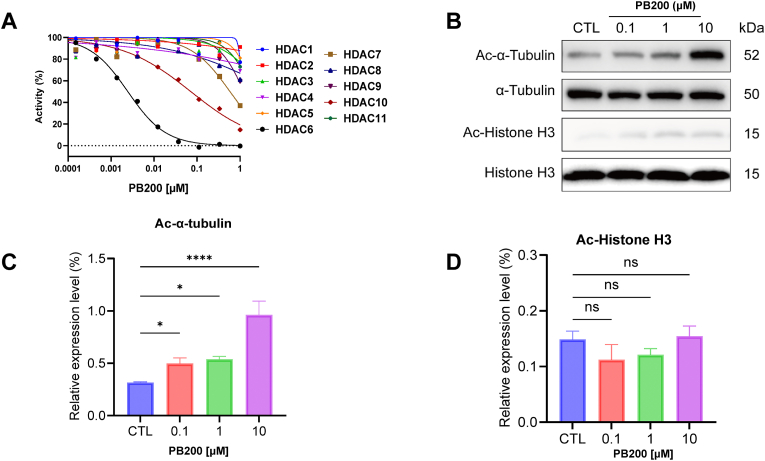
***In vitro* characterization of PB200.** (A) Comparative selectivity analysis of PB200 against multiple HDAC isoforms showing >141-fold selectivity for HDAC6. (B) Western blot analysis of acetylated α-tubulin and acetylated histone H3 levels in SH-SY5Y cells following treatment with increasing concentrations of PB200 (0.1, 1, and 10 μM). (*C*–D) Quantification of acetylated α-tubulin (C) and acetylated histone H3 (D) levels normalized to controls, demonstrating dose-dependent increase in α-tubulin acetylation without significant effect on histone H3 acetylation (ns = not significant). Data are presented as mean ± SD, n = 3, ∗p-value <0.05, ∗∗p-value <0.01, ∗∗∗p-value <0.001, ∗∗∗∗p-value <0.0001 by two-tailed unpaired Student's t-test.

Further target binding selectivity assessments revealed that PB200 possessed excellent selectivity toward HDAC6 over other HDAC isoforms (selectivity ratio >141-fold, Fig. 2A). In cellular assays, PB200 significantly upregulated the acetylation levels of α-tubulin in SH-SY5Y cells in a dose-dependent manner, while exhibiting negligible effects on Histone H3 acetylation (Fig. 2B–D). These enzymatic and cellular experimental findings collectively confirm the high affinity and selectivity of PB200 for HDAC6.

### 2.3 Metabolic stability and BBB permeability of PB200

Next, we evaluated the *in vitro* metabolic stability and BBB permeability of PB200. The compound demonstrated favorable metabolic stability in both mouse liver microsomes and hepatocytes, with half-lives (t_1/2_) of 58.2 min and 41.8 min, respectively (Table 2). To assess BBB permeability, mice were administered PB200 at a dose of 1 mg/kg via intravenous tail injection, and concentrations in brain tissue and plasma were determined at 0.5, 1-, and 4-h post-injection. The results revealed brain-to-plasma ratios (B/P) of 27.6, 16.4, and 4.6 at the respective time points, indicating excellent BBB penetration capability of the compound.

**Table 2:** ADME/PK studies of PB200^a^.

*In vitro* stability	
liver microsomal stability (mouse, t_1/2_, min)	58.2
hepatocyte stability (mouse, t_1/2_, min)	41.8

*In vivo* brain/plasma PK studies					
route	dose (mg/kg)	time (h)	brain concn (ng/mL)	plasma concn (ng/mL)	brain/plasma ratio
i.v	1	0.5	72.2	2.62	27.6
i.v	1	1	19.3	1.18	16.4
i.v	1	4	1.02	0.22	4.6

a The pharmacokinetic studies were performed by HD Biosciences (China) Co., Ltd. For the assessment of brain and plasma distribution, male C57BL/6 mice received PB200 via intraperitoneal (IP) administration at a dose of 1 mg/kg. Biological samples from both brain tissue and blood were harvested at designated time intervals (0.5, 1, and 4 h post-administration). Sample preparation involved acetonitrile precipitation, followed by quantitative analysis using liquid chromatography-tandem mass spectrometry (LC-MS/MS). All reported values represent the mean of three independent experimental replicates.

### 2.4 Radiosynthesis of [^18^F]PB200

**Scheme 1:**
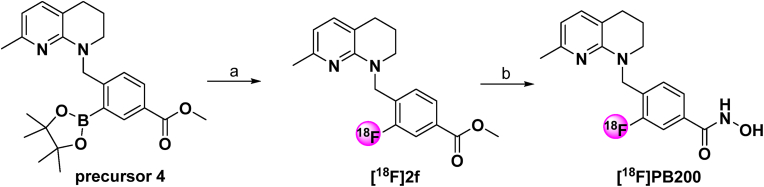
**Radiosynthesis of [^18^F]PB200**. Reagents and conditions: (a) K_222_/[^18^F]KF, Cu(OTf)_2_(Py)_4_, DMA, 110 °C, 20 min; (b) 0.6 M NaOH, NH_2_OH (50 wt % in H_2_O), MeOH/THF (1:1), 10 min.

We conducted radiolabeling of PB200 with fluorine-18 at the 3-position of the phenyl linker. As illustrated in Scheme 1, the radiofluorination was achieved using boronic ester precursor **4** under copper-mediated radiochemical conditions to obtain the intermediate [^18^F]**2f**. This intermediate was subsequently subjected to hydroxylamine hydrolysis to yield [^18^F]PB200. The total preparation time for [^18^F]PB200 was 70–80 min, with radiochemical yield (RCY) of 13 ± 4 % (n = 6, decay corrected). The final product demonstrated excellent radiochemical purity (>98 %) and high molar activity (127 ± 8 GBq/μmol, n = 6).

### 2.5 Autoradiography of [^18^F]PB200

**Fig. 3:**
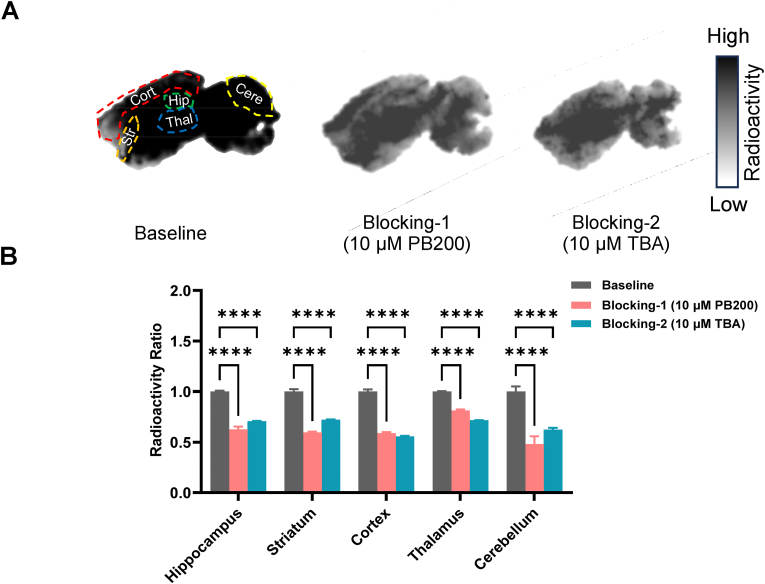
***In vitro* autoradiography of [^18^F]PB200 in mouse brain tissue.** (A) Representative autoradiographs showing differential distribution of [^18^F]PB200 across various brain regions under baseline conditions, with highest accumulation in hippocampus, cortex, and striatum. (B) Quantitative analysis of [^18^F]PB200 binding in different brain regions under baseline conditions and following competitive inhibition with 10 μM unlabeled PB200 or Tubastatin A (TBA), demonstrating specific binding of the radiotracer. Data are presented as mean ± SD, n = 3, ∗p-value <0.05, ∗∗p-value <0.01, ∗∗∗p-value <0.001, ∗∗∗∗p-value <0.0001 by two-tailed unpaired Student's t-test.

After obtaining [^18^F]PB200, we conducted autoradiography experiments on mouse brain slices to evaluate its distribution in brain tissue and binding specificity. Under baseline conditions, [^18^F]PB200 exhibited differential distribution across various brain regions in mouse brain tissue, with higher radioactivity observed in the hippocampus, cortex, and striatum (Fig. 3A). In the blocking experiments, we added 10 μM of unlabeled PB200 or the known HDAC6 inhibitor Tubastatin A (TBA) to the buffer used for incubating the brain tissue (Fig. 3B). The results showed that the radioactive signals in all brain regions were significantly reduced after the addition of the blocking agents, demonstrating the binding specificity of [^18^F]PB200.

### 2.6 Evaluation of [^18^F]PB200 in rodents using dynamic PET imaging

**Fig. 4:**
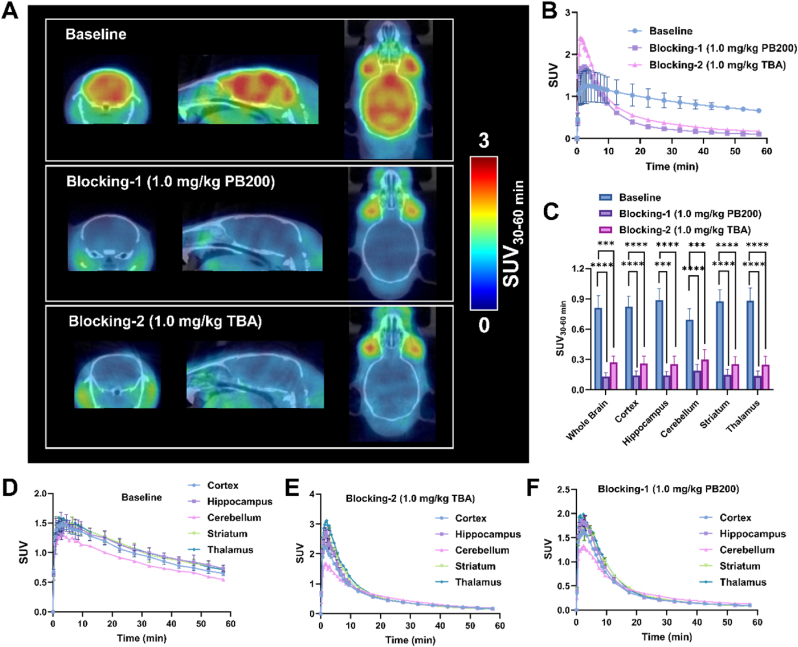
**PET imaging evaluation of [^18^F]PB200 in rodents. (**A) Representative PET/CT images (30–60 min post-injection) showing brain distribution of [^18^F]PB200 under baseline conditions and following pretreatment with PB200 (1.0 mg/kg) or Tubastatin A (1.0 mg/kg). (B) Time-activity curves demonstrating radiotracer accumulation (baseline) and blocking effects in mouse whole brain. (C) SUVs of baseline and blocking show [^18^F]PB200 uptake in cortex, hippocampus, cerebellum, striatum, and thalamus. (D–F) Comparative time-activity curves of [^18^F]PB200 in different brain regions under baseline and blocking conditions. Data are presented as mean ± SD, n = 4. ∗p-value <0.05, ∗∗p-value <0.01, ∗∗∗p-value <0.001, ∗∗∗∗p-value <0.0001 by two-tailed unpaired Student's t-test.

*In vivo* PET imaging studies were performed to assess the distribution and binding characteristics of the novel radiotracer [^18^F]PB200. Male rodents (n = 4) underwent dynamic PET acquisition for 60 min following intravenous administration. Quantitative analysis of integrated PET/computed tomography (CT) data acquired during the 30–60 min interval exhibited substantial cerebral accumulation of the radiotracer, with maximum standardized uptake values (SUV) reaching 1.4 (Fig. 4A and B). Volumetric assessment of radioactivity distribution demonstrated distinct regional variations across cerebral structures. Quantitative analysis revealed enhanced accumulation within limbic structures, particularly the hippocampal formation, striatal complex, and thalamic nuclei, while cerebellar regions exhibited diminished radiotracer concentration (Fig. 4D).

To validate target engagement specificity, competitive inhibition studies were conducted utilizing both the non-radioactive PB200 and Tubastatin A (TBA). Administration of either competing ligand preceding radiotracer injection resulted in marked reduction of [^18^F]PB200 accumulation across all regions of interest, indicating the *in vivo* binding specificity of [^18^F]PB200 (Fig. 4D–F).

In addition, we conducted vivo metabolite analysis of [^18^F]PB200, we examined the radiometabolic profile of [^18^F]PB200 in mice at 30 and 60 min postinjection. As result, [^18^F]PB200 demonstrated acceptable metabolic stability in brain tissue, with 85.2 ± 2.8 % and 67.4 ± 3.7 % of the parent compound remaining intact at 30 and 60 min, respectively (Fig. S3). In contrast, metabolism was significantly more extensive in plasma, where the intact fraction of [^18^F]PB200 decreased from 51.8 ± 3.3 % at 30 min to 21.2 ± 1.2 % at 60 min.

### 2.7 PET imaging of [^18^F]PB200 in NHPs

**Fig. 5:**
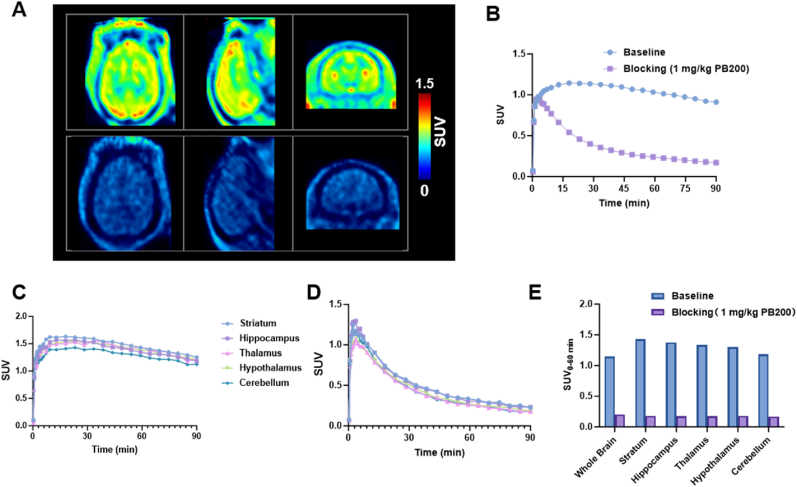
**NHP PET imaging of [^18^F]PB200.** (A) Summed PET images (axial, sagittal, and coronal views) from 30 to 90 min after injection of [^18^F]PB200 in a rhesus macaque at baseline (top) and after pre-treatment with 1 mg/kg of non-radioactive PB200 (blocking, bottom). (B) Time-activity curves demonstrating the kinetics of [^18^F]PB200 in whole brain under baseline conditions and blocking conditions. (C) Baseline time-activity curves for each region of interest in the brain. (D) Blocking time-activity curves for each region of interest in the brain. (E) Regional SUV analysis at 30–90 min post-injection across different brain regions showing substantial reduction in [^18^F]PB200 uptake following pre-treatment with unlabeled PB200.

Next, we performed PET imaging studies in non-human primates (NHPs). As shown in Fig. 5A, baseline scans demonstrated adequate uptake of [^18^F]PB200 across multiple brain regions. The time-activity curve demonstrated that [^18^F]PB200 has a rapid brain uptake that peaked around 15–20 min post-injection (SUV ∼1.2) and maintained relatively stable retention throughout the 90-min scanning period (Fig. 5B). In contrast, the blocking study using 1 mg/kg of unlabeled PB200 showed markedly reduced tracer uptake throughout the brain, indicating significant specific binding of our radiotracer. Regional analysis at 30–90 min post-injection (Fig. 5C and D) demonstrated that [^18^F]PB200 distributed heterogeneously across brain regions, with highest uptake in the striatum, followed by the hippocampus, thalamus, whole brain, hypothalamus, and cerebellum. The blocking study with unlabeled PB200 substantially reduced tracer uptake by approximately 80 % across all regions, with SUV_30–90 min_ falling below 0.20 (Fig. 5E). This results confirm the specificity of [^18^F]PB200 binding in the non-human primate brain.

### 2.8 PET imaging of [^18^F]PB200 in CUMS-induced MDD mouse model

**Fig. 6:**
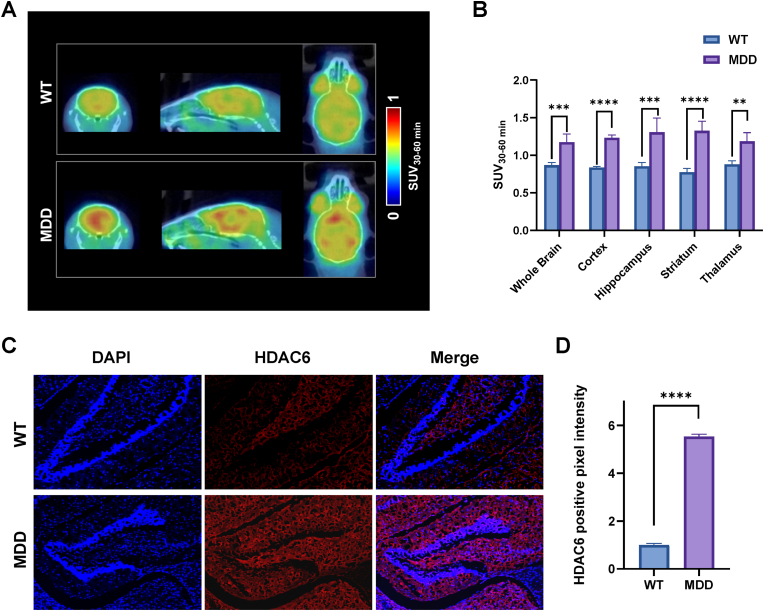
**PET imaging and immunofluorescence analysis of HDAC6 expression in a CUMS-induced depression mouse model.** (A) Representative PET images showing increased [^18^F]PB200 uptake in brains of MDD mice compared to WT controls. B) Quantitative analysis of [^18^F]PB200 regional uptake in MDD and control mice across multiple brain regions, with significant increases observed in regions of interest. C) Representative immunofluorescence images of hippocampus sections from HC and MDD mice. Brain sections were stained with DAPI (blue) to visualize cell nuclei and anti-HDAC6 antibody (red) to detect histone deacetylase 6 expression. The merged images demonstrate substantially increased HDAC6 immunoreactivity throughout the hippocampus in MDD compared to HC. D) Quantitative analysis of HDAC6 expression in WT and MDD mice. Data are presented as mean ± SD, n = 8, MDD = major depression disorder, ∗p-value <0.05, ∗∗p-value <0.01, ∗∗∗p-value <0.001, ∗∗∗∗p-value <0.0001 by two-tailed unpaired Student's t-test.

Following the successful validation of [^18^F]PB200 in mouse and NHP studies, we investigated its utility in examining HDAC6 expression patterns in MDD using CUMS-induced mouse model. Comparative dynamic PET imaging studies were performed using [^18^F]PB200 in both MDD and age-matched wild-type (WT) mice (n = 8 per group). Quantitative analysis of PET data revealed significantly elevated radiotracer accumulation in the brains of MDD mice compared to HC (Fig. 6A and B). To investigate the region-specific alterations in HDAC6 expression, we conducted detailed analyses of [^18^F]PB200 distribution across distinct brain regions. Notably, MDD mice displayed enhanced radiotracer uptake across all regions of interest, with particularly pronounced differences in the hippocampus, striatum, and amygdala—structures critically involved in learning, memory, and cognitive processes. Furthermore, we conducted *in vitro* experiments (immunofluorescence). As shown in Fig. 6C and D, the immunofluorescence experiment indicated significant upregulation of HDAC6 expression in the hippocampal region of MDD mice. Our *in vitro* assays demonstrated a significant increase in HDAC6 in the pathological brain of MDD mice, consistent with our PET imaging results.

### 2.9 Detection of neuroinflammation in CUMS-induced depression models

**Fig. 7:**
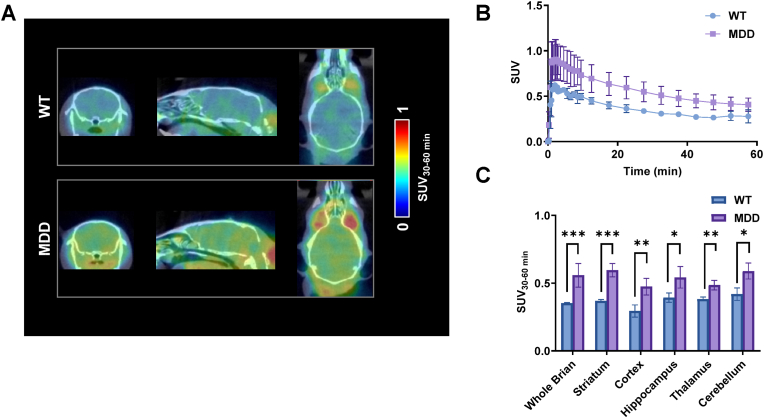
**TSPO-targeted PET imaging of neuroinflammation in CUMS-induced depression model.**(A) Representative [ ^18^F]FEPPA PET images in CUMS and WT mice. (B) Quantitative analysis of [^18^F]FEPPA regional uptake in CUMS and control mice across multiple brain regions. Data are presented as mean ± SD, n = 8, MDD = major depression disorder, ∗p-value <0.05, ∗∗p-value <0.01, ∗∗∗p-value <0.001 by two-tailed unpaired Student's t-test.

Previous investigations have established a significant correlation between HDAC6 and neuroinflammation, wherein HDAC6 upregulation potentiates neuroinflammatory responses, including microglial activation and pro-inflammatory cytokine release [8,[32], [33], [34]]. In the present study, we conducted *in vitro* and *in vivo* studies to characterize the neuroinflammatory profile in CUMS-induced depression model mice. Firstly, we employed PET imaging utilizing [^18^F]FEPPA [35], a well-characterized radioligand targeting translocator protein (TSPO), to explore the neuroinflammation in the CUMS model. PET imaging was performed on both HC and CUMS-induced MDD model mice. The results demonstrated significantly heightened radiotracer uptake in multiple brain regions of MDD mice relative to HC (Fig. 7A–C), suggesting that neuroinflammation occurs under MDD conditions.

**Fig. 8:**
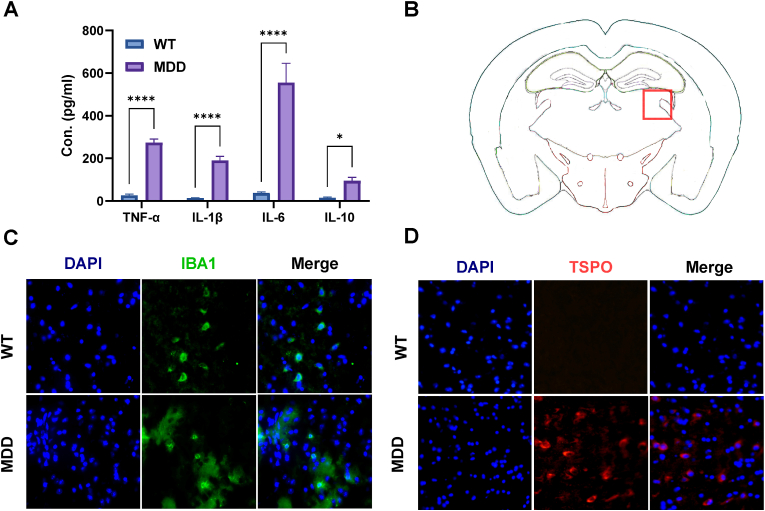
**Neuroinflammatory changes in CUMS-induced depression model.** (A) Quantification of pro-inflammatory cytokines (TNF-α, IL-1β, IL-6) and anti-inflammatory cytokine (IL-10) levels in brain tissue from MDD and HC mice. (B) Coronal brain section indicating the hippocampal region (red box) from which tissue was taken for analysis. (C) Representative images showing increased expression of the microglial marker IBA1 (green) in the hippocampus of MDD mice. (D) Immunofluorescence staining for the neuroinflammation marker TSPO (red) was markedly upregulated in MDD mice compared to HCs. Cell nuclei were counterstained with DAPI (blue). Scale bar = 50 μm. Data are presented as mean ± SD, n = 3, MDD = major depression disorder, ∗p-value <0.05, ∗∗p-value <0.01, ∗∗∗p-value <0.001 by two-tailed unpaired Student's t-test.

To investigate the inflammatory status of the MDD model, we first measured peripheral cytokine levels. We observed a significant elevation in the serum concentrations of pro-inflammatory cytokines TNF-α, IL-1β, and IL-6, as well as the anti-inflammatory cytokine IL-10, in MDD mice compared to WT (Fig. 8A). To determine if this peripheral inflammation was mirrored in the central nervous system, we performed immunofluorescence analysis on brain tissue. The analysis revealed a marked increase in the expression of IBA1, a marker for microglial activation, in the hippocampus of MDD mice (Fig. 8C). Furthermore, we observed a strong upregulation of the TSPO in the MDD group, whereas its expression was minimal in WT mice (Fig. 8D). These findings provide compelling evidence of a robust neuroinflammatory phenotype, characterized by both systemic cytokine elevation and central glial activation, in the MDD mouse model.

## 3 Discussion

HDAC6 has demonstrated a strong association with MDD, positioning it as a promising therapeutic target. However, current understanding of HDAC6's role in depression has been predominantly limited to *in vitro* experiments and animal behavioral studies. To address this research gap, HDAC6-targeted PET imaging enables monitoring of expression changes in living organisms, offering new insights into depression mechanisms and potential treatment approaches.

Our preliminary work focused on developing HDAC6 PET probes with high target specificity and effective BBB permeability. In our early-stage development of HDAC6 selective inhibitors, we conducted extensive structure-activity relationship studies on HDAC6 inhibitors and established the structure-activity relationships (SAR) for the Cap group, linker, and zinc binding group (ZBG) [31,36,37]. The Cap interacts with HDAC6's hydrophobic pocket, and strategic modifications to this region can enhance both selectivity for HDAC6 and improve BBB penetration through optimized lipophilicity. Based on these structural principles, we identified the tetrahydronaphthyridine group as an effective Cap structure that provides both potency and selectivity for HDAC6 with reasonable brain uptake. Our initial compound, PB131, while promising, demonstrated insufficient BBB penetration for neurological applications. This led us to focus on optimizing its naphthyridine Cap by introducing specific lipophilic groups to enhance the molecule's physiochemical properties and BBB permeability. This systematic approach resulted in PB200, which exhibits nanomolar affinity for HDAC6, high selectivity against other HDAC isoforms, and significantly improved pharmacokinetic properties compared to both PB131 and our previous HDAC6 PET probe ligand PB118 [36].

For practical clinical application, we optimized the radiolabeling methodology for [^18^F]PB200, employing copper-mediated radiofluorination of a boronic ester precursor followed by hydroxylamine hydrolysis. This streamlined two-step procedure represents a significant advancement over the more complex three-step synthesis required for [^18^F]Bavarostat [38], offering practical advantages for routine radiopharmaceutical production, including simplified purification, higher radiochemical yields, and reduced synthesis time. Moreover, PB200 demonstrated superior inhibitory activity and selectivity against HDAC6 at the enzymatic level *in vitro* compared to Bavarostat, which is more favorable for the radiotracer's *in vivo* specificity. With [^18^F]PB200 available, we conducted comprehensive evaluation of [^18^F]PB200 as an HDAC6 imaging agent through both *in vitro* autoradiography and *in vivo* PET imaging studies. Our results confirmed the target engagement of [^18^F]PB200, which showed preferential accumulation in the hippocampus, cortex, and striatum, regions with established high HDAC6 expression in rodents. In NPH imaging we also observed heterogeneous signal distribution and a blocking effect. Although [^18^F]PB200 showed good metabolic stability in mice and no defluorination was detected, it is noteworthy that in monkey brain images, radioactivity accumulation is observed around the brain. We speculate that this may be due to bone uptake resulting from defluorination, and possibly related to physiological uptake in the highly vascularized craniofacial muscles. Future studies should consider increasing the number of large animals and further testing its stability. Although *in vivo* imaging results showed the specificity of [^18^F]PB200, many HDAC6 inhibitors, including Tubastatin A, were repoted to bind to MBLAC2 [39], resulting the off-target effects. As we did not assess PB200's affinity for MBLAC2, this interaction cannot be ruled out. Interestingly, our cross-species validation revealed kinetic differences. Under blocking conditions, rodents showed a rapid peak and washout, whereas NHPs exhibited a uniform uptake reduction from the outset. These discrepancies likely stem from species-specific variations in blood-brain barrier permeability, transporter expression, and metabolism. Therefore, future kinetic modeling of [^18^F]PB200 must account for these species-specific factors to improve the translational relevance of its use in depression research. Following these successful validations, we applied [^18^F]PB200 to investigate HDAC6 distribution and expression in depression pathology. The imaging findings were corroborated by *ex vivo* analyses confirming HDAC6 upregulation and decreased α-tubulin acetylation in depression models. This study provides the first *in vivo* molecular imaging evidence directly linking increased HDAC6 activity with depressive pathophysiology, establishing [^18^F]PB200 as a valuable tool for investigating HDAC6's role in depression.

Neuroinflammation plays a crucial role in depression pathology, as evidenced by significantly elevated pro-inflammatory cytokines (IL-1β, IL-6, and TNF-α) in MDD [8,33]. These inflammatory signals activate brain microglia, triggering neuroinflammation that disrupts neurotransmitter metabolism, synaptic plasticity, and neurodegeneration, ultimately leading to depressive behaviors. Our previous research has established that HDAC6 is abnormally upregulated in various neurological conditions, including Alzheimer's disease [36,40], inflammation [31], and cerebral ischemia [41]. To explore the specific relationship between depression, neuroinflammation, and HDAC6, we conducted *in vitro* experiments and TSPO-targeted PET imaging studies using the CUMS-induced depression model. PET imaging with [^18^F]FEPPA revealed a significantly elevated TSPO signal in MDD mice, indicating microglial activation. While early-phase uptake could be influenced by perfusion changes, the signal remained elevated throughout the 60-min scan, suggesting genuine TSPO upregulation rather than a mere delivery artifact. This interpretation is strongly supported by our immunofluorescence data, which demonstrates increased TSPO, microglial activation (IBA1), and enhanced HDAC6 expression in MDD brain tissue. The co-localization of HDAC6 with IBA1 suggests that HDAC6-mediated epigenetic changes are a key mechanism driving this neuroinflammatory response. Future studies using perfusion-corrected modeling could further isolate the specific TSPO signal, refining our understanding of its relationship with HDAC6 overexpression in depression. These findings align with mounting evidence of neuroinflammation's role in depression pathogenesis and provide additional support that HDAC6 serves as a key regulatory factor in neuroinflammation-mediated depression. HDAC6 inhibition, showing promise in various neurological conditions, might have therapeutic potential in MDD by modulating microglial activation and subsequent inflammatory responses.

Several limitations of the present study should be noted. Our reliance on a semi-quantitative analysis was necessitated by the absence of a true reference region for HDAC6, which precluded kinetic modeling without invasive arterial sampling. While sufficient for demonstrating target engagement, this approach does not provide absolute quantification of receptor density. From a translational chemistry perspective, the catalyst-assisted synthesis, while efficient, presents a hurdle for widespread clinical adoption. The use of copper requires stringent, validated purification protocols and mandatory quality control testing for residual metal to meet regulatory standards for human use. These challenges are important considerations for the future clinical development of [^18^F]PB200.

## 4 Conclusions

Collectively, we have developed [^18^F]PB200, a selective, brain-penetrant HDAC6 PET tracer with nanomolar potency and favorable stability, produced via an efficient radiosynthesis. Across rodents and non-human primates, it demonstrated specific, displaceable binding *in vitro* and *in vivo*. PET imaging with [^18^F]PB200 revealed elevated HDAC6 in MDD, which co-localized with neuroinflammation markers and activated microglia, suggesting a mechanistic link. [^18^F]PB200 is a valuable tool for mapping HDAC6 to assess target engagement and monitor therapies, with clear potential for clinical translation to advance precision medicine in depression.

## 5 Experimental

Chemical reagents and solvents were obtained from commercial suppliers at ACS-grade purity or higher and used without additional purification. Tubastatin A was sourced from MedChemExpress. ^1^H NMR and ^13^C NMR spectra data were recorded on a JEOL JNM–ECZ500R Spectrometer at 400 MHz. Chemical shifts were given in *δ* values (ppm), using tetramethylsilane (TMS) as the internal standard or other residual internals including CDCl_3_ (*δ* 7.26), DMSO‑*d*_6_ (*δ* 2.50); coupling constants (*J*) were given in Hz. Signal multiplicities were characterized as s (singlet), d (doublet), t (triplet), q (quartet), dd (doublet of doublets), m (multiplet), and br (broad signal). Analytical thin layer chromatography (TLC) was performed on silica gel GF254. Column chromatographic purification was carried out using silica gel. Mass spectrometry data were recorded on an Agilent 6310 ion trap mass spectrometer (ESI source) connected to an Agilent 1200 series HPLC with quaternary pump, vacuum degasser, diode–array detector, and autosampler. All the final compounds were >95 % pure by HPLC. All animal studies were approved by the Institutional Animal Care and Use Committees (IACUC) at West China Hospital and Massachusetts General Hospital, following the guidelines of the US National Institutes of Health.

### 5.1 Synthesis of HDAC6 inhibitors

**General procedures for the preparation of intermediates 2ã2g.** To a stirred solution of the appropriate secondary heterocyclic amine (1.0 equiv) in dry THF at −78 °C was added NaHMDS (2.0 M in THF, 1.2 equiv) dropwise. After 30 min at −78 °C, a solution of methyl 4-(bromomethyl)-3-fluorobenzoate (1.2 equiv) in THF (2 mL per 10 mL reaction volume) was added slowly, and the mixture was maintained at −78 °C for 30–50 min before warming to room temperature and stirring until complete (monitored by TLC/LC–MS). The reaction was quenched with saturated NH_4_Cl, and the mixture was extracted with ethyl acetate. The combined organic layers were washed with brine, dried, filtered, and concentrated. The crude residue was purified by flash column chromatography, eluting with a gradient of ethyl acetate in petroleum ether (0–15 % EtOAc), to afford intermediates **2a**–**2g**.

**Procedure for the preparation of intermediates 2h.** A suspension of 7-chloro-3,4-dihydro-1H-1,8-naphthyridin-2-one (1.0 equiv) in dry DMF was cooled to 0 °C, and NaH (60 % dispersion in mineral oil, 1.2 equiv) was added portionwise under nitrogen. The mixture was stirred for 30 min at room temperature. The reaction was recooled to 0 °C and a solution of methyl 4-(bromomethyl)-3-fluorobenzoate (1.2 equiv) in DMF was added dropwise. The mixture was stirred at room temperature for 30–120 min until complete. The reaction was quenched with saturated NH_4_Cl, diluted with water, and extracted with ethyl acetate. The combined organic layers were washed with brine, dried, filtered, concentrated, and purified by flash chromatography (eluting with a gradient of ethyl acetate in petroleum ether (0–20 % EtOAc) to give intermediate **2h**.

**General procedures for the preparation of products 3ã3h.** In a cooled (0 °C) methanolic solution, hydroxylamine hydrochloride (30 equiv) was neutralized in situ with LiOH (23 equiv) in MeOH (5 mL per 25 mL flask) and stirred for 30 min at room temperature to generate free NH_2_OH. A solution of the corresponding methyl ester intermediate (**2a**–**2h**) in MeOH was added, and the reaction was stirred at room temperature for 30 min (monitored by TLC/LC–MS). After acidification to pH 6–7 with 4 M HCl, the residue was purified by reverse-phase flash chromatography (5–80 % MeOH/H_2_O gradient) to yield the final products **3a**–**3h**.

**Methyl-4-((7-chloro-3,4-dihydro-1,8-naphthyridin-1(2*H*)-yl)methyl)-3-fluor-obenzoate (2a).** Light yellow solid, yield: 60 %. ^1^H NMR (400 MHz, CDCl_3_): *δ* 7.78 (d, *J* = 8.0 Hz, 1H), 7.72 (d, *J* = 8.0 Hz, 1H), 7.47 (t, *J* = 8.0 Hz, 1H), 7.08 (d, *J* = 8.0 Hz, 1H), 6.49 (d, *J* = 8.0 Hz, 1H), 4.92 (s, 2H), 3.93 (t, *J* = 6.0 Hz 2H), 2.73 (t, *J* = 6.0 Hz 2H), 1.94 (m, 2H). MS (ESI) for C_17_H_16_ClFN_2_O_2_ [M + H]^+^ calcd 335.1, found: 335.2.

**Methyl-4-((6–chloro-3,4-dihydro-1,8–naphthyridin-1(2*H*)-yl)methyl)-3- fluor-obenzoate. (2b).** White solid, yield: 85 %. ^1^H NMR (400 MHz, CDCl3): *δ* 7.89 (d, *J* = 2.3 Hz, 1H), 7.77–7.70 (m, 2H), 7.34 (t, *J* = 8.0 Hz, 2H), 7.15 (m, 1H), 4.94 (s, 2H), 3.93 (s, 3H), 3.42–3.39 (m, 2H), 2.80–2.77 (m, 2H), 2.00–1.96 (m, 2H). MS (ESI) for C_17_H_16_ClFN_2_O_2_ [M + H]^+^ calcd 335.1, found: 335.2.

**Methyl-3-fluoro-4-((6-fluoro-3,4-dihydro-1,8–naphthyridin-1(2*H*)-yl)-methyl)-benzoate (2c)**. Colorless oil, yield: 83 %. ^1^H NMR (400 MHz, CDCl_3_): *δ* 7.81 (m, 1H), 7.76–7.70 (m, 2H), 7.35 (t, *J* = 8.0 Hz, 2H), 7.00 (d, *J* = 8.0 Hz, 1H), 4.93 (s, 2H), 3.93 (s, 3H), 3.39 (t, *J* = 4.0 Hz, 2H), 2.81 (t, *J* = 8.0 Hz, 2H), 2.01–1.97 (m, 2H). MS (ESI) for C_17_H_16_F_2_N_2_O_2_ [M + H]^+^ calcd 319.1, found: 319.4.

**Methyl-4- ((6-bromo-3, 4-dihydro-1,8-naphthyridin-1(2*H*)-yl)-methyl)-3-fluorobenzoate (2d)**. Light yellow oil. yield: 50 %. ^1^H NMR (400 MHz, CDCl_3_): *δ* 7.97 (m, 1H), 7.76–7.70 (m, 2H), 7.26 (m, 1H), 4.93 (s, 2H), 3.93 (s, 3H), 3.41–3.38 (m, 2H), 2.80–2.77 (m, 2H), 1.98–1.95 (m, 2H). MS (ESI) for C_17_H_16_BrFN_2_O_2_ [M + H]^+^ calcd 379.0, found: 379.2.

**Methyl-3-fluoro-4-((7-methyl-3,4-dihydro-1,8-naphthyridin-1(2*H*)-yl)methyl)-benzoate. (2e).** Light yellow oil, yield: 60 %. ^1^H NMR (400 MHz, CDCl_3_): *δ* 7.76–7.70 (m, 2H), 7.46 (t, *J* = 6.0 Hz, 1H), 7.05 (d, *J* = 8.0 Hz, 1H), 6.36 (d, *J* = 8.0 Hz, 1H), 4.99 (s, 2H), 3.93 (s, 3H), 3.38 (t, *J* = 6.0 Hz, 2H), 2.74 (t, *J* = 6.0 Hz, 2H), 2.33 (s, 3H), 1.98–1.93 (m, 2H). MS (ESI) for C_18_H_19_FN_2_O_2_ [M + H]^+^ calcd 315.2, found: 315.1.

**Methyl-4-((2-chloro-5,6,7,8-tetrahydro-9*H*-pyrido[2,3-*b*]azepin-9-yl)-methyl)-3-fluorobe-nzoate (2f).** Light yellow solid, yield: 45 %. ^1^H NMR (400 MHz, CDCl_3_): *δ* 7.79 (d, *J* = 8.0 Hz, 1H), 7.72 (d, *J* = 8.0 Hz, 1H), 7.53 (t, *J* = 8.0 Hz, 1H), 7.22 (d, *J* = 8.0 Hz, 1H), 6.66 (d, *J* = 8.0 Hz, 1H), 4.80 (s, 2H), 3.94 (s, 3H), 3.29 (s, *J* = 4.0 Hz, 2H), 2.77 (t, *J* = 4.0 Hz, 2H), 1.77 (m, 4H). MS (ESI) for C_18_H_18_FN_2_O_2_ [M + H]^+^ calcd 314.1, found: 314.2.

**Methyl-3-fluoro-4-((5,6,7,8-tetrahydro-9*H*-pyrido[2,3-*b*]-azepin-9-yl)-methyl)-benzoate (2g).** Light white oil, yield: 30 %. ^1^H NMR (400 MHz, CDCl_3_): *δ* 8.05 (d, *J* = 4.0 Hz, 1H), 7.81 (m, 1H), 7.71 (m, 1H), 7.50 (t, *J* = 8.0 Hz 1H), 7.33 (d, *J* = 8.0 Hz 1H), 6.73–6.99 (m, 1H), 4.83 (s, 2H), 3.93 (s, 3H), 3.24–3.21 (m, 2H), 2.83–2.80 (m, 2H), 1.80–1.74 (m, 4H). MS (ESI) for C_17_H_16_ClFN_2_O_2_ [M + H]^+^ calcd 335.1, found: 335.2. MS (ESI) for C_18_H_17_ClFN_2_O_2_ [M + H]^+^ calcd 348.1, found: 348.4.

**Methyl4-((6-chloro-2-oxo-3,4-dihydro-1,8-naphthyridin-1(2*H*)-yl)methyl)-3-fluoroben-zoate (2h).** White solid, yield: 80 %. ^1^H NMR (400 MHz, CDCl_3_): *δ* 8.13 (d, *J* = 4.0 Hz, 1H), 7.71–7.69 (m, 2H), 7.50–7.49 (m, 1H), 7.16 (t, *J* = 8.0 Hz, 1H), 5.45 (s, 2H), 3.91 (s, 3H), 3.00–2.97 (m, 2H), 2.84–2.80 (m, 2H). MS (ESI) for C_17_H_14_ClFN_2_O_2_ [M + H]^+^ calcd 349.1, found: 349.2.

**4-((7-chloro-3,4-dihydro-1,8-naphthyridin-1(2*H*)-yl)methyl)-3-fluoro-*N*-hydroxybenza-mide (3a)**. White solid, yield: 92 %.^1^H NMR (400 MHz, DMSO‑*d*_6_): *δ* 11.27 (m, 1H), 9.13 (s, 1H), 7.56–7.54 (m, 2H), 7.32 (t, *J* = 8.0 Hz, 1H), 7.24 (d, *J* = 8.0 Hz, 1H), 6.51 (d, *J* = 8.0 Hz, 1H), 4.79 (s, 2H), 3.40 (t, *J* = 8.0 Hz 2H), 2.71 (t, *J* = 8.0 Hz, 2H), 1.87 (m, 2H). ^13^C NMR (100 MHz, DMSO‑*d*_6_) *δ* 163.2, 161.9, 159.4, 155.3, 146.0, 138.8, 134.0, 134.0, 129.7, 129.6, 128.9, 128.7, 123.3, 116.3, 114.3, 114.1, 110.9, 48.0, 45.6, 45.6, 26.6, 20.9. HRMS (ESI) for C_16_H_16_ClFN_3_O_2_ [M + H]^+^ calcd 336.0910, found: 336.0901.

**4-((6-chloro-3,4-dihydro-1,8-naphthyridin-1(2*H*)-yl)methyl)-3-fluoro-*N*-hydroxybenza-mide (3b).** White solid, yield: 95 %. ^1^H NMR (400 MHz, DMSO‑*d*_6_): *δ* 11.26 (s, 1H), 9.12 (s, 1H), 7.82 (d, *J* = 2.0 Hz, 1H), 7.55–7.51 (m, 2H), 7.33 (s, 1H), 7.25 (s, *J* = 8.0 Hz, 1H), 4.83 (s, 2H), 3.42 (t, *J* = 5.2 Hz, 2H),2.77 (t, *J* = 6.0 Hz, 2H), 1.89 (m, 2H). ^13^C NMR (100 MHz, DMSO‑*d*_6_) *δ* 163.3, 161.8, 159.3, 154.0, 143.5, 135.3, 133.8, 133.7, 129.3, 129.2, 129.1, 123.3, 119.4, 118.0, 114.3, 114.0, 48.2, 45.7, 45.6, 27.2, 20.9. HRMS (ESI) for C_16_H_16_ClFN_3_O_2_ [M + H]^+^ calcd 336.0910, found: 336.0918.

**3-fluoro-4-((6-fluoro-3,4-dihydro-1,8-naphthyridin-1(2*H*)-yl)methyl)-*N*-hydroxybenza-mide (3c)**. White solid, yield: 90 %. ^1^H NMR (400 MHz, DMSO‑*d*_6_): *δ* 11.22 (s, 1H), 9.12 (s, 1H), 7.79 (d, *J* = 2.4 Hz, 1H), 7.54–7.50 (m, 2H), 7.27–7.25 (m, 2H), 4.81 (s, 2H), 3.93 (t, *J* = 2.4 Hz, 2H), 2.79 (t, *J* = 6.0 Hz, 2H), 1.90 (m, 2H). ^13^C NMR (100 MHz, DMSO‑*d*_6_) *δ* 163.2, 161.8, 159.4, 154.1, 152.3, 151.7, 133.8, 133.7, 131.6, 131.4, 129.5, 129.4, 129.3, 129.3, 124.2, 124.0, 123.2, 118.9, 118.8, 114.2, 114.0, 48.3, 45.8, 45.8, 27.4, 21.1. HRMS (ESI) for C_16_H_16_F_2_N_3_O_2_ [M + H]^+^ calcd 320.1205, found: 320.1213.

**4-((6-bromo-3,4-dihydro-1,8-naphthyridin-1(2*H*)-yl)methyl)-3-fluoro-*N*-hydroxybenza-mide (3d)**. White solid; yield: 92 %.^1^H NMR (400 MHz, DMSO‑*d*_6_): *δ* 11.27 (s, 1H), 9.14 (s, 1H), 7.88 (d, *J* = 2.0 Hz, 1H), 7.55–7.51 (m, 2H), 7.42 (d, *J* = 2.0 Hz, 1H), 7.24 (t, *J* = 8.0 Hz, 1H),4.82 (s, 2H), 3.40 (t, *J* = 5.6 Hz, 2H), 2.77 (t, *J* = 6.0 Hz, 2H), 1.89 (m, 2H). ^13^C NMR (100 MHz, DMSO‑*d*_6_) *δ* 163.1, 161.8, 159.3, 154.2, 145.7, 137.7, 133.9, 129.3, 129.2, 129.1, 129.0, 123.3, 120.1, 114.2, 114.0, 105.8, 48.2, 45.6, 45.6, 27.2, 20.9. HRMS (ESI) for C_16_H_16_BrFN_3_O_2_ [M + H]^+^ calcd 380.0404, found: 380.0415.

3-**fluoro-*N*-hydroxy-4-((7-methyl-3,4-dihydro-1,8-naphthyridin-1(2*H*)-yl)methyl)benza-mide (3e).** White solid; yield: 92 %. ^1^H NMR (400 MHz, DMSO‑*d*_6_): *δ* 11.25 (s, 1H), 9.12 (s, 1H), 7.53 (d, *J* = 9.2 Hz, 2H), 7.33 (t, *J* = 7.6 Hz, 1H), 7.08 (d, *J* = 7.2 Hz, 1H), 6.34 (d, *J* = 7.2 Hz, 1H), 4.85 (s, 2H), 3.36 (m, 2H), 2.69 (t, *J* = 6.0 Hz, 2H), 2.19 (s, 3H), 1.87 (m, 2H). ^13^C NMR (100 MHz, DMSO‑*d*_6_) *δ* 163.2, 161.9, 159.5, 154.7, 153.6, 136.5, 133.7, 133.6, 129.9, 129.8, 129.7, 123.2, 114.2, 114.0, 113.9, 111.4, 48.1, 45.0, 45.0, 27.1, 24.4, 21.6. HRMS (ESI) for C_17_H_19_FN_3_O_2_ [M + H]^+^ calcd 316.1456, found: 316.1462.

**5-((2-chloro-5,6,7,8-tetrahydro-9*H*-pyrido[2,3-*b*]azepin-9-yl)methyl)-3-fluoro-*N*-hydro-xybenzamide (3f).** White solid, yield: 93 %.^1^H NMR (400 MHz, DMSO‑*d*_6_): *δ* 11.27 (s, 1H), 9.13 (s, 1H), 7.56–7.52 (m, 2H), 7.44–7.36 (m, 2H), 6.69 (d, *J* = 8.0 Hz, 1H), 4.68 (s, 2H), 3.37 (s, 2H), 2.76 (s, 2H),1.73 (s, 4H). ^13^C NMR (100 MHz, DMSO‑*d*_6_) *δ* 163.2, 162.0, 160.2, 159.5, 145.2, 141.9, 133.9, 133.8, 130.4, 130.4, 129.8, 129.7, 123.5, 123.2, 114.2, 114.0, 113.7, 50.7, 48.5, 31.1, 27.8, 24.6. HRMS (ESI) for C_17_H_18_ClFN_3_O_2_ [M + H]^+^ calcd 350.1066, found: 350.1068.

**3-fluoro-*N*-hydroxy-4-((5,6,7,8-tetrahydro-9*H*-pyrido[2,3-*b*]azepin-9-yl)methyl)benza-mide (3g)**. White solid, yield: 90 %.^1^H NMR (400 MHz, DMSO‑*d*_6_): *δ* 11.25 (s, 1H), 9.11 (s, 1H), 7.93 (d, *J* = 4.0 Hz, 1H), 7.53–7.50 (m, 2H), 7.41–7.36 (m, 2H), 6.68 (t, *J* = 8.0 Hz, 1H), 4.72 (s, 2H), 3.62 (s, 2H), 2.77 (s, 2H), 1.71 (s, 4H). ^13^C NMR (100 MHz, DMSO‑*d*_6_) *δ* 163.3, 161.9, 160.7, 159.5, 145.0, 138.8, 133.6, 133.5, 130.7, 130.6, 130.2, 130.1, 125.5, 123.1, 115.4, 114.1, 113.9, 51.4, 48.6, 48.6, 32.6, 28.5, 25.01. HRMS (ESI) for C_17_H_19_FN_3_O_2_ [M + H]^+^ calcd 316.1456, found: 316.1454.

**4-((6-chloro-2-oxo-3,4-dihydro-1,8-naphthyridin-1(2*H*)-yl)methyl)-3-fluoro-*N*-hydroxy-benzamide (3h).** White solid; yield: 90 %. ^1^H NMR (400 MHz, DMSO‑*d*_6_): *δ* 11.25 (s, 1H), 9.12 (s, 1H), 8.19 (s, 1H), 7.87 (s, 1H), 7.52 (d, *J* = 11.2 Hz, 1H), 7.45 (d, *J* = 8.0 Hz, 1H), 7.12 (t, *J* = 8.0 Hz, 1H), 5.26 (s, 2H), 3.02 (t, *J* = 8.0 Hz, 2H), 2.78 ppm (t, *J* = 8.0 Hz, 2H). ^13^C NMR (100 MHz, DMSO‑*d*_6_) *δ* 170.6, 163.1, 161.2, 158.8, 150.5, 144.2, 136.3, 133.8, 133.7, 128.4, 128.4, 128.3, 125.4, 123.5, 123.2, 114.1, 113.9, 38.2, 38.1, 30.6, 22.9. HRMS (ESI) for C_16_H_14_ClFN_3_O_3_ [M + H]^+^ calcd 350.0702, found: 350.0694.

### 5.2 *In vitro* HDAC inhibitory assays

The HDAC enzyme inhibition activity assay was conducted by Reaction Biology Corporation, San Diego, CA. The HDAC enzyme inhibition activity was determined using a fluorogenic substrate-based assay. Test compounds were dissolved in DMSO and serially diluted, followed by addition of 10 μL of test compound solution to each well. Subsequently, 80 μL of HDAC enzyme solution prepared in Tris-HCl buffer (pH 8.0 containing 0.1 % BSA) was added. The mixture was incubated at room temperature for 15 min to allow potential inhibitors to bind to the enzyme. The reaction was initiated by adding 10 μL of isoform-specific fluorogenic substrates: HDACs 1, 2, 3, and 6 utilized fluorogenic peptide from p53 residues 379–382 (RHKK(Ac)AMC); HDACs 4, 5, 7, 9, and 11 employed fluorogenic HDAC Class2a Substrate (Trifluoroacetyl Lysine); HDAC8 used fluorogenic peptide from p53 residues 379–382 (RHK(Ac)K(Ac)AMC); and HDAC10 utilized Ac-Spermidine-AMC. The reaction mixtures were incubated at 37 °C for 45 min. The reaction was terminated and fluorescence developed by adding 50 μL of developer solution, followed by further incubation at room temperature for 20 min. Fluorescence intensity was measured using a microplate reader at excitation and emission wavelengths of 355 nm and 460 nm, respectively. Inhibition percentage was calculated as: [1-(Test compound RFU-Blank RFU)/(Control RFU-Blank RFU)] × 100. IC_50_ values were determined by non-linear regression analysis of the inhibition percentage versus compound concentration curve. All experiments were performed in triplicate.

### 5.3 Cellular assays and western blot analysis

SH-SY5Y neuroblastoma cells were maintained in DMEM supplemented with 10 % fetal bovine serum and 1 % penicillin–streptomycin. The cells were treated with various concentrations of PB200 (0.1, 1, and 10 μM) for 24 h. After treatment, the cells were harvested, washed twice with phosphate-buffered saline (PBS), and centrifuged to remove the supernatant. Cell lysis was performed using RIPA buffer (Beyotime) containing phenylmethylsulfonyl fluoride (PMSF), a protease inhibitor cocktail, and a phosphatase inhibitor cocktail (Sigma). The lysates were incubated on ice for 4 min, followed by gentle rotation for 1 min to ensure complete cell lysis. Subsequently, the samples were intermittently vortexed and incubated on ice for 30 min. After centrifugation at 13,800×*g* for 15 min at 4 °C, the supernatant was collected as the total protein extract. Protein concentration was determined using a BCA protein assay kit (Beyotime). Equal amounts of protein were separated by SDS-polyacrylamide gel electrophoresis (SDS-PAGE) and transferred onto methanol-activated polyvinylidene fluoride (PVDF) membranes. The membranes were blocked with 5 % skim milk in Tris-buffered saline containing 0.1 % Tween-20 (TBST) for 2 h at room temperature to prevent nonspecific binding. Thereafter, the membranes were incubated with primary antibodies overnight at 4 °C. After washing with TBST, the membranes were incubated with horseradish peroxidase (HRP)-conjugated secondary antibodies for 1–2 h at room temperature, followed by additional TBST washes. Protein bands were visualized using an enhanced chemiluminescence (ECL) detection kit (Abbkine) and imaged with a chemiluminescence imaging system.

The following antibodies were used: Acetyl-α-Tubulin Rabbit mAb (ABclonal, A24738) and α-Tubulin Rabbit mAb (ABclonal,AC049). Histone H3 Rabbit mAb (ABclonal,A17562). Acetyl-Histone H3–K4/K9/K14/K18/K23/K27 Rabbit pAb (ABclonal, A21295).

### 5.4 Radiochemistry

Radiosynthesis of [^18^F]PB200: Refer to the previous method [36], aqueous [^18^F]fluoride from the cyclotron target was trapped on a Waters Sep-Pak Light QMA carbonate cartridge, which had been pre-conditioned sequentially with ethanol (10 mL), 1 M K_2_CO_3_ (10 mL), and sterile water (10 mL). The trapped [^18^F]fluoride was then eluted into a reaction vial using 1.0 mL of a stock solution containing Kryptofix 222 (10 mg) and K_2_CO_3_ (0.5 mg) in acetonitrile/water (4/1, v/v). The solvent was subsequently removed by azeotropic distillation under a gentle nitrogen stream at 100 °C. 1.0 mL of anhydrous acetonitrile was added and the drying process was repeated twice to ensure moisture removal. Next, a solution of the boronic ester precursor (compound 4, 2.0 mg) and the catalyst Cu(OTf)_2_ (py)_4_ (5 mg) in DMA (500 μL) was added to the vial containing the dried [^18^F]fluoride complex. The reaction mixture was stirred at 110 °C for 20 min to form the intermediate, [^18^F]**2f**. After cooling to room temperature, the reaction was quenched with HPLC mobile phase and the intermediate was purified by semi-preparative reverse-phase HPLC (Column: Agilent Eclipse XDB-C18, 5 μm, 250 mm × 9.4 mm, flow rate = 4.0 mL/min, mobile phase = 0.1 % TFA in water/0.1 % TFA in acetonitrile, 25/75, v/v). The HPLC fraction containing the purified intermediate [^18^F]**2f** was collected and loaded onto a pre-conditioned C18 Sep-Pak cartridge. [^18^F]**2f** was then subjected to hydrolysis directly on the cartridge by passing a solution of 50 % hydroxylamine (NH_2_OH) in a methanolic sodium hydroxide solution (1 mL) through the cartridge. After 10 min at room temperature, the resulting mixture was injected into a reverse-phase semipreparative HPLC for purification (Column: Agilent Eclipse XDB-C18, 5 μm, 250 mm × 9.4 mm, flow rate = 4.0 mL/min, mobile phase = 0.1 % TFA in water/0.1 % TFA in acetonitrile, 60/40, v/v). The final product, [^18^F]PB200, was eluted from the C18 cartridge with ethanol (1 mL) and subsequently formulated in a solution of 10 % ethanol in sterile saline (v/v) for *in vitro* and *in vivo* studies. The total synthesis time, from end-of-bombardment (EOB) to the final formulated product, was approximately 70–80 min. The decay-corrected radiochemical yield was 13 ± 4 % (n = 6), with a radiochemical purity of >98 % and a molar activity of 127 ± 8 GBq/μmol (n = 6) at the end of synthesis.

Radiosynthesis of [^18^F]FEPPA [42]: The precursor and standard compound were synthezed as the route shown in Scheme S2. For the radiosynthesis of [^18^F]FEPPA, the tosylate precursor (**1**, 2 mg in 500 μL acetonitrile) was reacted with K_2_CO_3_/K_222_ complex at 90 °C for 10 min, followed by TFA quenching. Purification employed reverse-phase semipreparative HPLC on an Agilent Eclipse XDB-C18 column using water/acetonitrile (73:27 v/v) containing 0.1 % TFA at 4.0 mL/min. The radiochemical yield ranged from 24 ± 3 % (decay corrected, n = 3) with ≥98 % radiochemical purity and specific activity of 188 ± 4 GBq/μmol at end of synthesis.

The probes’ validation was performed by co-injection of radioactive probe and non-labeled standard compound in analytical HPLC (Fig. S1-S2).

### 5.5 *In vitro* autoradiography

The *in vitro* autoradiography experiment was referred to our previously reported method with minor modifications [43]. Brain tissues from wild-type mice were harvested, flash-frozen, and sectioned (20 μm thickness) using a cryostat. Brain sections were incubated with [^18^F]PB200 (0.74 MBq/mL) in PBS containing 0.1 % BSA for 60 min at room temperature. For blocking studies, sections were co-incubated with [^18^F]PB200 and either unlabeled PB200 (10 μM) or Tubastatin A (10 μM). After incubation, sections were washed with cold PBS, dried, and exposed to a phosphor imaging plate. Autoradiographs were acquired using a Typhoon FLA 7000 phosphor imager and analyzed using ImageJ software to quantify regional radioactivity.

### 5.6 Radiometabolite analysis of [^18^F]PB200

The *in vivo* metabolic stability of [^18^F]PB200 was assessed in male C57BL/6 mice. Following a tail-vein injection of approximately 17 MBq of the radiotracer, separate animal cohorts (n = 3 per group) were used for brain and plasma analysis at 30 and 60 min post-injection. For plasma analysis, trunk blood was collected, and plasma was separated via centrifugation. A plasma aliquot was then deproteinized using ice-cold acetonitrile and centrifuged, after which the supernatant was filtered (0.22 μm). For brain analysis, mice first underwent transcardial perfusion. The brain was then excised, homogenized in an acetonitrile/saline solution, and the homogenate was centrifuged and filtered. Both processed plasma and brain samples were analyzed by an analytical radio-HPLC system. An Alltech Chrom BDS column was used with a mobile phase of 50/50 acetonitrile/water with 0.1 % formic acid, at a flow rate of 5 mL/min. The percentage of intact, unmetabolized [^18^F]PB200 was determined by integrating the peak area corresponding to the parent compound in the radio-chromatogram. This provided a quantitative measure of the radiotracer's stability in both the central nervous system and the periphery.

### 5.7 CUMS-induced depression model

Male C57BL/6 mice (19–22 g) were acclimated for 5 days under standard conditions (23 °C, 12-h light/dark cycle) with ad libitum access to food and water before experimentation. Mice developing illness during the study were excluded. All behavioral assessments were conducted by a single experimenter blinded to group assignments to minimize variability. Following acclimation, CUMS mice were individually housed and subjected to one daily random stressor for four weeks. Stressors included: 24-h food or water deprivation, 24-h wet bedding exposure, 24-h housing in bedding-free cages, 3-h tube restraint, plantar electric shock (0.4 mA, 2-s duration with 0–30 s intervals) for 22 min, and 3-h cold water (4 °C) immersion. Control animals remained group-housed under standard conditions without stressors. After four weeks, depression-like behaviors were evaluated using four behavioral tests. In the Forced Swim Test, immobility time was measured during the final 4 min of a 6-min water immersion. For the Tail Suspension Test, mice were suspended by their tails and immobility was recorded during the last 4 min of a 6-min period. Open Field Test assessed locomotion and anxiety by tracking movement and center time in a 50 × 50 × 50 cm arena for 5 min. In the Novelty-Suppressed Feeding Test, food-deprived mice were placed in a novel environment with food, and latency to feeding was measured with a 6-min maximum. Behavioral experiment results are shown in Fig. S4.

### 5.8 *In vivo* PET imaging in rodents

Rodents PET imaging studies were conducted by referring to our previous research methodology [44,45]. All animal experiments were conducted using a small animal PET/CT scanner (Inveon, Siemens) with mice under 2 % isoflurane anesthesia. For baseline HDAC6 imaging, male C57BL/6 mice (n = 4, 8–10 weeks old) received a tail vein injection of [^18^F]PB200 (7.4–11.1 MBq), followed by a 60-min dynamic PET scan. For blocking studies, separate cohorts were pre-treated with either unlabeled PB200 (1.0 mg/kg) or Tubastatin A (1.0 mg/kg) 5 min before the radiotracer injection. Pathological PET imaging was conducted in a CUMS model of depression and matched controls. The WT and MDD groups each included 8 mice (4 males, 4 females). Animals were scanned with [^18^F]PB200 using the same acquisition parameters as above. To assess neuroinflammation, [^18^F]FEPPA PET was performed in both HC and MDD groups under identical acquisition conditions; when both tracers were used in the same animal, scans were separated by at least 48 h. For all PET scans, data were reconstructed using a 3D OSEM/MAP algorithm and co-registered with CT images for anatomical reference. The analysis of detailed distribution of different brain regions of mice was obtained through the mouse (Ma-Benveniste-Mirrione) VOI atlas, and time-activity curves were generated to quantify regional uptake as standardized uptake values (SUV).

### 5.9 NHP PET/CT imaging

The PET/CT imaging studies in NHPs in this work were referred to our previous work [46,47]. Briefly, Male rhesus macaques (weight: 10.5 kg) underwent 12-h fasting before imaging. Anesthesia was induced with intramuscular Zoletil 50 (tiletamine-zolazepam, 0.05 mL/kg) and maintained with 3 % pentobarbital sodium (15 mg/kg). Brain imaging was performed on a Discovery PET/CT 710 scanner. Dynamic PET acquisition began immediately before [^18^F]PB200 administration (average: 5.07 mCi), with anatomical CT images acquired for attenuation correction and anatomical reference. For blocking studies, unlabeled PB200 (1.0 mg/kg) was administered 5 min pre-radiotracer injection. PET data were recorded in list mode, corrected for attenuation, scatter, and decay, then reconstructed into progressively longer time frames. Images were converted to SUV by normalizing for injected dose and subject weight. The analysis of detailed distribution of different brain regions was obtained through the INIA19 primate brain atlas.

### 5.10 Immunohistochemistry and western blot analysis

After anesthesia and cardiac perfusion, mouse brain tissues were collected and post-fixed. The tissues were dehydrated sequentially in 15 %, 20 %, and 30 % sucrose solutions prepared in phosphate-buffered saline (PBS) at 4 °C until they sank, indicating complete cryopreservation. Subsequently, the tissues were embedded in OCT compound (SAKURA Tissue-Tek® OCT Compound) and sectioned using a cryostat to a thickness of 10–20 μm. The resulting sections were stored at −80 °C until further use. For immunofluorescence staining, frozen sections were first equilibrated at room temperature for 3–5 min. They were then washed three times with PBS, 10 min each, to remove residual embedding medium and rehydrate the tissue. A hydrophobic barrier pen (ZSBG-BIO, ZLI-9305) was used to outline the tissue boundaries to prevent reagent spread. The sections were then covered completely with 5 % normal goat serum and placed in a humidified chamber for blocking. After removal of the blocking solution, diluted primary antibody was applied, and the sections were incubated overnight at 4 °C. The following day, the primary antibody solution was removed, and the sections were rinsed once with PBST for 5 min, followed by three additional 5-min washes with PBST. Subsequently, the sections were incubated with diluted secondary antibody for 1 h at room temperature in the dark. After removal of the secondary antibody, the sections were washed once with PBST for 5 min and then three times with PBST, 5 min each. Finally, the sections were washed once with TBST for 5 min and three times with TBS, 5 min each. We quantified the average fluorescence intensity within the region of interest using ImageJ software. For each experimental group, at least three independent tissue sections were analyzed. The data are presented as the mean ± standard error of the mean (SEM) derived from all region of interest measurements per group.

The primary antibodies used included HDAC6 Rabbit pAb (ABclonal, A11259), PBR/TSPO Rabbit mAb (ABclonal, A4881), and AIF1/IBA1 Rabbit mAb (ABclonal, A19776). These antibodies were employed to detect HDAC6 expression and the microglial marker IBA1, respectively, in brain tissues from both control and model mice.

### 5.11 Neuroinflammation assessment

The concentrations of cytokines in brain tissue homogenates from CUMS and control mice were quantified using enzyme-linked immunosorbent assay (ELISA) kits, following the manufacturers’ instructions. The following kits from Beyotime (China) were used:Mouse IL-6 ELISA Kit (PI326), Mouse TNF-α ELISA Kit (PT513), Mouse IL-1β ELISA Kit (PI301), Mouse IL-10 ELISA Kit (PI522). These assays were employed to assess the levels of the pro-inflammatory cytokines IL-6, TNF-α, and IL-1β, as well as the anti-inflammatory cytokine IL-10, in order to evaluate neuroinflammatory responses associated with the CUMS model.

## Funding source

This research was supported by grants from Basic Scientific Research Funds for 10.13039/100007231Sichuan University (20826041H4046), Sichuan Provincial Natural Science Foundation (25NSFSC2196), General Program of Sichuan Science and Technology Program (2025ZNSFSC0657), and 1.3.5 Project for Disciplines of Excellence, 10.13039/501100013365West China Hospital, Sichuan University (137220092, ZYGD23016).

## CRediT authorship contribution statement

**Yanting Zhou:** Data curation, Formal analysis. **Yuheng Zou:** Data curation, Methodology, Writing – review & editing. **Xiao Zhong:** Data curation, Methodology. **Hongyan Li:** Formal analysis. **Jingyi Yang:** Methodology. **Hui Meng:** Methodology. **Weiyao Xie:** Data curation. **Pan Yao:** Data curation. **Xiaoai Wu:** Data curation. **Huawei Cai:** Resources. **Lin Li:** Resources. **Changning Wang:** Conceptualization, Funding acquisition, Investigation. **Wei Zhang:** Conceptualization, Funding acquisition, Investigation. **Ping Bai:** Conceptualization, Funding acquisition, Investigation, Methodology, Project administration.

## Declaration of competing interest

The authors declare they have no competing interests.

## Data Availability

Data will be made available on request.
