# Supplementary material for: Development of a novel HDAC6 PET imaging agent uncovers associations between HDAC6 overexpression and neuroinflammation in depression
Source: Redox Biol. 2026 Jan 12;90:104014. doi: 10.1016/j.redox.2026.104014 (PMC12834932; doi:10.1016/j.redox.2026.104014)
Supplement: Multimedia component 1 [file mmc1.docx]

**Supporting Information**

**Development of a Novel HDAC6 PET Imaging Agent Uncovers Associations Between HDAC6 Overexpression and Neuroinflammation in Depression**

Yanting Zhou^1,2,3,4,5#^, Yuheng Zou^6#^, Xiao Zhong^6,7#^, Hongyan Li^1,2,3,4,5^, Jingyi Yang^8^, Hui Meng^1,2,3,4,5^, Weiyao Xie^1,2,3,4,5^, Pan Yao^1,2,3,4,5^, Xiaoai Wu^6^, Huawei Cai^6^, Lin Li^6^, Changning Wang^9^*, Wei Zhang^8^*, Ping Bai^1,2,3,4,5^*

^1^Department of Pulmonary and Critical Care Medicine, West China Hospital, State Key Laboratory of Respiratory Health and Multimorbidity, Sichuan University, Chengdu, Sichuan, 610041, China

^2^Molecularly Targeted Research and Development Laboratory, Institute of Respiratory Health, Frontiers Science Center for Disease-related Molecular Network, West China Hospital, Sichuan University, Chengdu, Sichuan, 610041, China

^3^Precision Medicine Center, Precision Medicine Key Laboratory of Sichuan Province, West China Hospital, Sichuan University, Chengdu, Sichuan, 610041, China

^4^The Research Units of West China, Chinese Academy of Medical Sciences, West China Hospital, Chengdu, Sichuan, 610041, China

^5^Institute of Respiratory Health and Multimorbidity, West China Hospital, Sichuan University, Chengdu, Sichuan, 610041, China

^6^Department of Nuclear Medicine, Laboratory of Clinical Nuclear Medicine, West China Hospital, Sichuan University, Chengdu, Sichuan, 610041, China

^7^West China Biomedical Big Data Center, West China Hospital, Sichuan University, Chengdu, Sichuan, 610041, China

^8^Mental Health Center and Psychiatric Laboratory, the State Key Laboratory of Biotherapy, West China Hospital, Sichuan University, Chengdu, Sichuan, 610041, China

^9^Athinoula A. Martinos Center for Biomedical Imaging, Department of Radiology, Massachusetts General Hospital, Harvard Medical School, Charlestown, MA 02129, United States

*Corresponding authors. Tel./fax: +1 617-724-3983

Email address: [cwang15@mgh.harvard.edu](mailto:cwang15@mgh.harvard.edu) (C.W.); [weizhanghx@163.com](mailto:weizhanghx@163.com) (W.Z.); [pingbai@scu.edu.cn](mailto:pingbai@scu.edu.cn) (P.B.)

**Table of Contents**

[**Scheme S1. Synthesis of the radiolabeling precursor of [^18^F]PB200** 3](#_Toc218709319)

[**Scheme S2. Synthesis of the standard compound and radiolabeling precursor of [^18^F]FEPPA** 5](#_Toc218709320)

[**Figure S1**. **The HPLC chromatogram of [^18^F]PB200 and PB200** 8](#_Toc218709321)

[**Figure S2. The HPLC chromatogram of [^18^F]FEPPA and FEPPA** 9](#_Toc218709322)

[**Figure S3. metabolic stability assay of [^18^F]PB200 in mouse blood and brain.** 10](#_Toc218709323)

[***In vitro* metabolic stability evaluation of PB200** 10](#_Toc218709324)

[**Mice brain/plasma PK studies of PB200** 11](#_Toc218709325)

[**Figure S4. Behavioral experimental results of the CUMS-Induced MDD Model** 12](#_Toc218709326)

[**NMR Spectrum of synthetic compounds** 13](#_Toc218709327)

[**References** 23](#_Toc218709328)

NMR spectra were collected in a JEOL NMR-ECZ500R Spectrometer at room temperature (500 MHz (^1^H), 471 MHz (^19^F), and 126 MHz (^13^C)). Chemical shifts were given in *δ* values (ppm), using tetramethylsilane (TMS) as the internal standard. Mass spectrometry data were recorded on an Agilent 6310 ion trap mass spectrometer (ESI source) connected to an Agilent 1200 series HPLC with a quaternary pump, vacuum degasser, diode-array detector, and autosampler.

# **Scheme S1. Synthesis of the radiolabeling precursor of [^18^F]PB200**

**Reagents and conditions**: (a) NBS, CCl_4_, 70 °C; (b) NaHMDS, THF, -78°C; (c) Pd(dppf)Cl_2_, CH_2_Cl_2_, B_2_Pin_2_, KOAc, Dioxane, 110 °C

**Procedure of synthesis of methyl 3-bromo-4-(bromomethyl)benzoate (2).** To a mixture of methyl 3-bromo-4-methylbenzoate (4.0 g, 1 *eq*) in carbontetrachloride (100 mL) were added N-bromosuccinimide(3.7 g, 2 *eq*). The mixture was stirred at 70 °C overnight under nitrogen. The reaction mixture was then cooled to room temperature, quenched with 20 mL ofsaturated aqueous sodium bicarbonate solution, and extracted withdichloromethane (100 mL × 2). The combined organic layers were washed with 20mL of saturated brine, dried over anhydrous sodium sulfate, filtered andconcentrated under vacuum. The residue was purified by silica gel column chromatographyeluted with petroleum ether/ethyl acetate (v/v = 5/1), to give intermediate 2 as a white solid as a white solid.(4.61 g, 82% yield) .*^1^*H NMR (400 MHz, Chloroform-d) δ 8.24 (d, *J* = 1.6 Hz, 1H), 7.95 (dd, *J* = 8.0, 1.6 Hz, 1H), 7.53 (d, *J* = 8.0 Hz, 1H), 4.60 (s, 2H), 3.93 (s, 3H).

**Procedure of synthesis of Methyl 3-bromo-4-((7-methyl-3,4-dihydro-1,8-naphthyridin-1(2*H*)-yl)methyl)benzoate (3).** To a solution of 7-methyl-1,2,3,4-tetrahydro-1,8-naphthyridine （2 g, 1 *eq*）in THF ( 20 mL) was cooled to -78 °C. NaHMDS (2M) solution (1.2 *eq*) was added to the mixture and stirred at -78 °C . After stirring at low temperature for 30 minutes, slowly add the intermediate 2 (4.99 g,2 *eq*) dissolved in THF ( 5 mL). Continue the reaction at -78°C for 30-50 minutes after the addition is complete. Transfer to room temperature stirring. After reaction completion, remove solvent by distillation, dilute with ethyl acetate/water, extract, dry with saturated brine and sodium sulfate, then remove solvent under reduced pressure.he residue was purified by silica gel column chromatography eluted with petroleum ether/ethyl acetate (v/v =3/1), to give intermediate 3 as a yellow oil. (4.5 g, 89% yield).^1^H NMR (400 MHz, DMSO-d_6_) δ 8.11 (d, *J* = 1.6 Hz, 1H), 7.88 (dd, *J* = 8.0, 1.6 Hz, 1H), 7.28 (d, *J* = 8.0 Hz, 1H), 7.11 (d, *J* = 7.2 Hz, 1H), 6.35 (d, *J* = 7.2 Hz, 1H), 4.84 (s, 2H), 3.85 (s, 3H), 2.73 (t, *J* = 6.4 Hz, 2H), 2.13 (s, 3H), 1.96 – 1.86 (m, 2H).

**Procedure of synthesis of (5-(methoxycarbonyl)-2-((7-methyl-3,4-dihydro-1,8-naphthyridin-1(2*H*)-yl)methyl)phenyl)boronic acid (4).**To a solution of Intermediate 3 (200 mg,1 *eq*), Pd(dppf)Cl_2_.CH_2_Cl_2_ (43.52 mg,0.1 *eq*), B_2_Pin_2_ (406,02 mg,3 *eq*), and KOAc (156.92 mg.3 *eq*) were s*eq*uentially added to the reaction tube. After diluting with an appropriate amount of 1,4-dioxane solvent (3 mL）and purging with nitrogen, the mixture was heated at 110°C for 10 h. Upon completion, the solvent was removed under vacuum, the mixture was diluted with ethyl acetate and extracted. The organic phase was collected, concentrated under vacuum, and purified by column chromatography (petroleum ether: ethyl acetate = 2:1) to obtain (5-(methoxycarbonyl)-2-((7-methyl-3,4-dihydro-1,8-naphthyridin-1(2*H*)-yl)methyl)phenyl)boronic acid as a white solid.(78.78 mg, 35% yield).^1^H NMR (400 MHz, DMSO-d_6_) δ 8.65 (s, 2H), 8.11 (d, *J* = 2.0 Hz, 1H), 7.85 (dd, *J* = 8.0, 2.0 Hz, 1H), 7.33 (d, *J* = 8.0 Hz, 1H), 7.08 (d, *J* = 7.2 Hz, 1H), 6.31 (d, *J* = 7.2 Hz, 1H), 4.92 (s, 2H), 3.83 (s, 3H), 3.42 (t, *J* = 5.6 Hz, 2H), 2.69 (t, *J* = 6.4 Hz, 2H), 2.16 (s, 3H), 1.94 – 1.79 (m, 2H).^13^C NMR (101 MHz, DMSO-d6) δ 166.92, 154.99, 153.51, 149.81, 136.60, 134.77, 130.18, 127.32, 127.02, 114.41, 111.40, 52.39, 51.76, 49.18, 27.21, 24.02, 21.57.

# **Scheme S2. Synthesis of the standard compound and radiolabeling precursor of [^18^F]FEPPA**

**Reagents and conditions:** (a) Phenol, K_2_CO_3_, 70 °C, 2 h; (b) SnCl_2_, HCl, 65 °C, 16 h; (c) *o*-salicylaldehyde, NaBH_4_, MeOH, 90 °C~0 °C~r.t, 2 h; (d) Acetyl Choride, DMAP, LiOH, MeOH, r.t., 24 h; (e) 1,2-Bis(tosyloxy)ethane, NaH, DMF, r.t., 1 h; (f) 2-fluoroethyl tosylate, TBAOH, DMF, 40 °C, 16 h.

**Synthesis of** **4-Phenoxypyridin-3-amine (1)**

Stir a mixture of 4-chloro-3-nitropyridine (1.5g, 9.5 mmol), K_2_CO_3_ (2.3 g, 16.7 mmol), and phenol (1.2 g, 12.8 mmol) in DMF (20 mL) and heat it to 70°C for 2 hours. Then, continue stirring at room temperature overnight. Quench the reaction mixture with water (50ml) and extract it with ethyl acetate (2×50 mL). Combine the organic extracts and wash them with saturated K_2_CO_3_ aqueous solution, water, and brine. Dry the combined organic phase over Na_2_SO_4_ and filter it to remove the solvent, leaving behind a red oily substance. Treat the product with MeOH (100 mL), SnCl_2_ (13.4 g, 197 mmol), and 6N HCl (15 mL) and reflux for 16 hours. After vacuum evaporation of MeOH, adjust the residue to pH > 10 with 5N NaOH and extract it with EtOAc (2×100 mL). Combine the organic extracts and wash them with water and brine, then dry the organic phase over Na_2_SO_4_ and filter it. Remove the solvent under vacuum to obtain a colorless oil, which is then dried under high vacuum to yield compound **1**, 4-Phenoxypyridin-3-amine (1.55g, 87%).

**Synthesis of 2-((4-phenoxypyridin-3-ylamino)methyl)phenol (2)**

4-Phenoxy-pyridin-3-amine (1.55 g, 8.3 mmol) and ortho-salicylaldehyde (1.5 g, 12.5 mmol) were stirred and heated at 90°C for 1 hour. The mixture was then cooled to 0°C in an ice bath, followed by the addition of MeOH (20 mL), and then NaBH4 (0.85 g, 22.4 mmol) was added slowly in portions over 20 minutes. The ice bath was removed, and the mixture was stirred for an additional 30 minutes at room temperature. Subsequently, formic acid (0.6 mL) was added, and the stirring continued for 15 minutes. The volatile components were removed under reduced pressure, and the residue was mixed with EtOAc (60ml) and a saturated NaHCO_3_ aqueous solution (50 mL). The resulting precipitate was collected by vacuum filtration, washed with a small amount of EtOAc, and vacuum-dried to yield a white solid compound **2**, 2,2-((4-phenoxypyridin-3-ylamino)methyl)phenol (2.07 g, 85%). ^1^H NMR (400 MHz, DMSO-d_6_): δ = 9.79 (bs, 1H), 7.87 (s, 1H), 7.70 (d, *J* = 4.0 Hz ,1H), 7.46 (t, *J* = 8.0 Hz 1H), 7.24 (t, *J* = 8.0 Hz, 1H), 7.19 (d, *J* = 8.0 Hz, 1H), 7.12 (d, *J* = 8.0 Hz, 2H), 7.05 (t, *J* = 6.0 Hz, 1H), 6.83 (t, *J* = 8.0 Hz, 1H), 6.72 (t, *J* = 8.0 Hz, 1H), 5.93 (bs, 1H), 4.35 (s, 2H).

**Synthesis of *N*-(2-hydroxybenzyl)-*N*-(4-phenoxypyridin-3-yl) acetamide (3)**

The compound **2**, 2-((4-phenoxy-3-pyridinyl)aminomethyl)phenol (0.517 g, 1.77 mmol), and 4-dimethylaminopyridine (5 mg) were stirred in anhydrous DCM (5 mL), and a slow dropwise addition of 5 mL of a 17% acetyl chloride solution in DCM (v/v) was carried out. After 24 hours of stirring at room temperature, DCM was removed by vacuum evaporation. At room temperature, a solution of LiOH in MeOH (5ml) was added to the residue. After stirring for 10 minutes, water (10 mL) was added, and MeOH was removed on a rotary evaporator. The aqueous solution was extracted twice with EtOAc, and the combined extracts were washed with brine, dried (Na_2_SO_4_), and filtered, and the solvent was removed, leaving an oily solid. It was ground with hexane and then recrystallized from EtOAc/hexane to yield a white solid compound **3**, *N*-(2-hydroxybenzyl)-*N*-(4-phenoxy-3-pyridinyl)acetamide. ^1^H NMR (400 MHz, CDCl_3_): δ = 9.28 (s, 1H), 8.44 (s, *J* = 8.0 Hz, 1H), 8.34 (s , 1H), 7.41 (t, *J* = 8.0 Hz, 1H), 7.31-7.23 (m, 3H), 6.97 (d, *J* = 8.0 Hz, 1H), 6.80 (d, *J* = 8.0 Hz, 2H), 6.74 (t, *J* = 8.0 Hz, 1H), 6.69-6.65 (m, 2H), 4.84 (d, *J* = 4.0 Hz, 1H), 2.05 (s, 3H).

**Synthesis of 2-(2-((*N*-(4-phenoxypyridin-3-yl)acetamido)methyl)phenoxy)ethyl 4-methylbenzenesulfonate (Precursor 1)**

NaH ( 0.27 g, 60%, 6.2 mmol) was added to a dried flask in an oven, then, a solution of N,N-dimethylacetamide (3) (2.23 g, 6.67 mmol) in DMF (25 mL) was added, and the mixture was stirred at room temperature under a nitrogen atmosphere for 10 minutes. Subsequently, a solution of ethane-1,2-diyl bis(4-methylbenzenesulfonate) (recrystallized from chloroform, 3.5 g, 9.45 mmol) in DMF (15 mL) was added in one portion. The reaction mixture was stirred under nitrogen for 50 minutes, quenched with water (300 mL), and extracted with EtOAc twice. The collected organic phase was washed three times with water and brine, dried over MgSO_4_, and filtered. The solvent was removed under reduced pressure, leaving a brown solid (2.6 g). Gradient flash chromatography [using EtOAc:hexane (50:50), followed by 1-5% MeOH in EtOAc] was performed twice to yield a waxy solid (1.77g, 50%). A portion (0.89g) was recrystallized from a 95% ethanol-water solution to obtain white crystals of precursor **1,** 2-(2-((*N*-(4-phenoxypyridin-3-yl)acetamido)methyl)phenoxy)ethyl 4-methylbenzenesulfonate (0.59 g). ^1^H NMR (400 MHz, CDCl_3_): δ = 8.28 (s, 1H), 8.19 (s, *J* = 8.0 Hz, 1H), 7.80 (d , 2H), 7.46-7.40 (m, 3H), 7.35-7.29 (m, 3H), 6.92 (m, 3H), 6.66 (d, *J* = 8.0 Hz, 2H), 6.74 (t, *J* = 4.0 Hz, 1H), 5.06 (d, *J* = 16.0 Hz, 1H), 4.87 (d, *J* = 16.0 Hz, 1H), 4.25-4.11 (m, 2H), 4.00-3.91 (m, 2H), 2.45 (s, 3H), 2.00 (s, 3H).

**Synthesis of *N*-(2-(2-fluoroethoxy)benzyl)-*N*-(4-phenoxypyridin-3-yl)acetamide (standard compound, 4)**

A stirred solution of N-(2-hydroxybenzyl)-N-(4-phenoxy-3-pyridinyl)acetamide (**3**) (1.0 g, 3.0 mmol) in DMF (15 mL) was treated with 1N tetrabutylammonium hydroxide in MeOH (3 mL), followed by a slow dropwise addition of a solution of 2-fluoroethyl p-toluenesulfonate (0.90g, 4.1 mmol) in DMF (2 mL). After stirring at 40°C for 16 hours, water (100ml) was added, and the mixture was extracted with EtOAc twice. The combined extracts were washed with saturated K_2_CO_3_ aqueous solution and brine, dried over Na_2_SO_4_, and filtered. The solvent was removed, leaving behind a brown oily substance (1.03 g). Gradient flash chromatography [using EtOAc: hexane (50:50), followed by 1-5% MeOH in EtOAc] yielded the pure product as a viscous colorless oil, compound **4**, *N*-(2-(2-fluoroethoxy)benzyl)-*N*-(4-phenoxy-3-pyridinyl)acetamide (0.48 g, 42%). ^1^H NMR (400 MHz, CDCl_3_): δ = 8.31 (s, *J* = 8.0 Hz, 1H), 8.22 (s, 1H), 7.47-7.41 (m, 3H), 7.31 (d, *J* = 8.0 Hz, 1H), 7.24 (t, *J* = 8.0 Hz, 1H), 6.95-6.92 (m,, 3H), 6.76 (d, *J* = 8.0 Hz, 1H), 6.62 (d, *J* = 8.0 Hz, 1H), 5.26 (d, *J* = 16.0 Hz, 1H), 4.88 (s, *J* = 12.0 Hz, 1H), 4.69-4.44 (m, 2H), 4.13-3.93 (s, 2H), 2.01 (s, 3H).


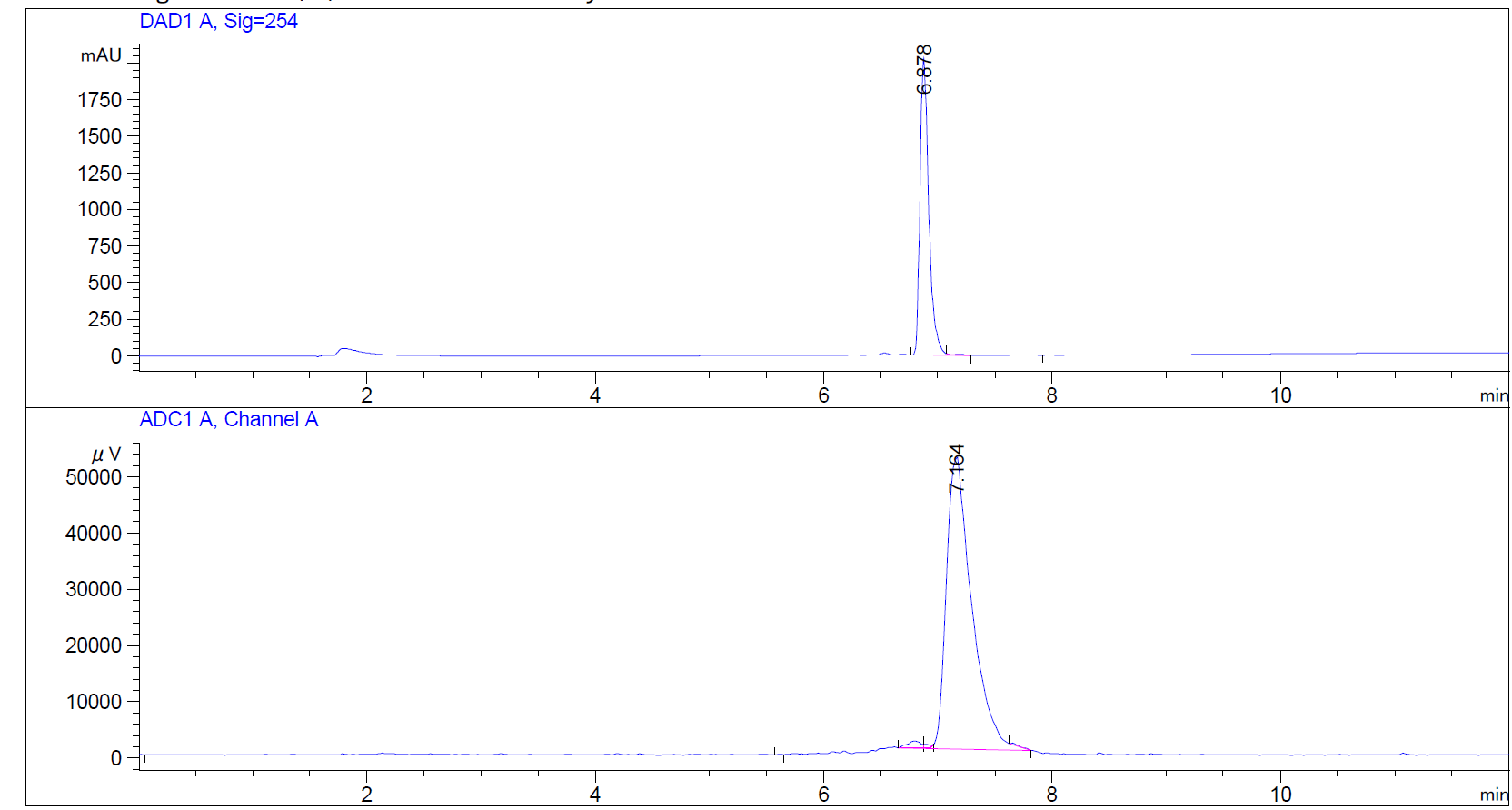


**Figure S1**. **The HPLC chromatogram of** **[^18^F]PB200 and PB200**. Analytic HPLC condition: Agilent Eclipse plus C18, 3.5 *μ*m, 4.6×100 mm, flow rate = 1.0 mL/min, mobile phase = 0.1% formic acid in water / 0.1% formic acid in acetonitrile, gradient method.


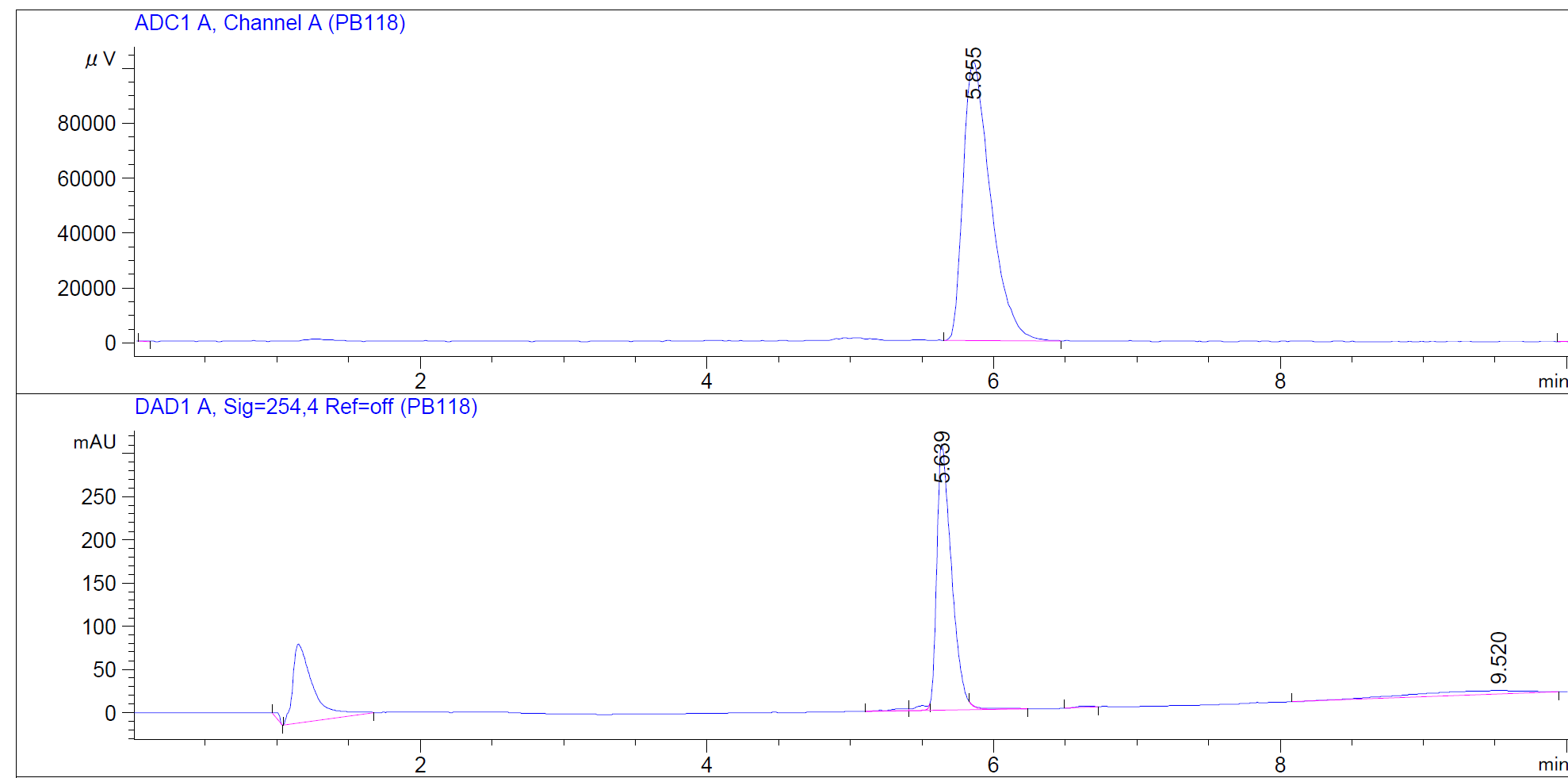


**Figure S2. The HPLC chromatogram of [^18^F]FEPPA and FEPPA**. Analytic HPLC condition: Agilent Eclipse plus C18, 3.5 *μ*m, 4.6×100 mm, flow rate = 1.0 mL/min, mobile phase = 0.1% formic acid in water / 0.1% formic acid in acetonitrile, gradient method.

***In Vivo* Radiometabolites Analysis.** The *in vivo* metabolic profile of [^18^F]PB8200 was assessed in male C57BL/6 mice (20-25 g). All animals received a bolus tail-vein injection of approximately 17 MBq of [^18^F]PB8200. Separate cohorts of mice were used for brain and plasma analysis (n = 3 per group per time point) at 30 and 60 minutes post-injection. For plasma analysis, mice were euthanized by cervical dislocation, and trunk blood was immediately collected into heparinized tubes. Plasma was separated by centrifugation (4,000 g, 5 min, 4°C). A 200 µL plasma aliquot was deproteinized with 400 µL of ice-cold acetonitrile, vortexed, and centrifuged (14,000 g, 5 min). The resulting supernatant was filtered (0.22 µm) for HPLC analysis. The HPLC system was equipped with Alltech Chrom BDS column (10µ, 10*250 mm) and eluted with a mobile phase of acetonitrile and H_2_O mixture containing 0.1% HCOOH (50/50, v/v) with a flow rate of 5 mL/min. For brain analysis, the animal was subjected to transcardial perfusion. Following perfusion, the brain was immediately excised, weighed, and homogenized in a 3:1 (v/w) solution of acetonitrile and saline (1:1). The homogenate was centrifuged (14,000 g, 10 min), and the supernatant was filtered (0.22 µm). Processed brain and plasma samples were analyzed by analytical HPLC equipped with radiometric and UV detectors (Agilent 1100 HPLC system with a BIOSCAN FC3200 flow counter). The percentage of intact [^18^F]PB8200 was determined by radio-HPLC chromatogram integration, comparing the peak corresponding to the parent compound.


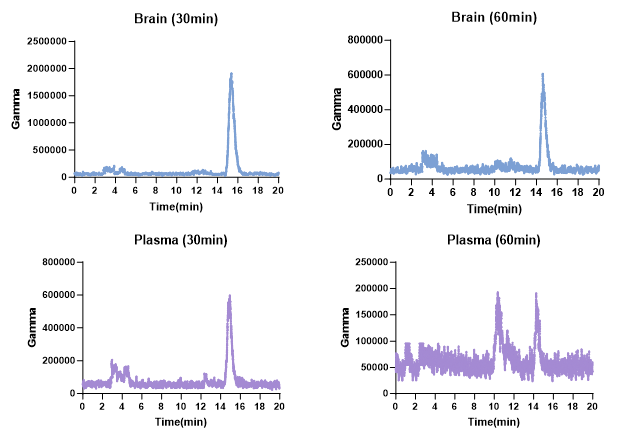


**Figure S3. metabolic stability assay of [^18^F]PB200 in mouse blood and brain.** Representative analytic-HPLC chromatograms illustrating the metabolic profile of [^18^F]PB200 in mouse brain and plasma at 30 and 60 min post-injection. The separation was performed using a gradient elution from 5% to 95% acetonitrile in water.

***In vitro* metabolic stability evaluation of PB200^1, 2^**

*In vitro* metabolic stability studies were conducted by HD Biosciences (China) Co., Ltd. Test compound PB200 was weighed and dissolved in 100% DMSO to create a 10 mM stock solution. **Liver Microsome Stability Assay:** The PB200 stock solution was diluted to 500 μM with 50% acetonitrile. Incubations were performed in duplicate in 96-well plates. Initial reaction mixtures contained 40 µL of 0.1 M potassium phosphate buffer (pH 7.4), 4.125 mM MgCl_2_, 0.625 mg/mL liver microsomes, and either test compound (1.25 µM) or a positive control. After a 5-minute preincubation at 37 °C, the enzymatic reaction was initiated by adding 10 µL of 5.0 mM NADPH in 0.1 M potassium phosphate buffer. This resulted in final concentrations of: 0.1 M potassium phosphate buffer (pH 7.4), 1.0 mM NADPH, 3.3 mM MgCl_2_, 0.5 mg/mL liver microsomes, and 1.0 µM of test compound or positive control. Reactions were stopped at 0, 5, 10, 20, and 40 minutes by adding 200 µL of ice-cold acetonitrile containing an internal standard. A negative control, substituting 0.1 M potassium phosphate buffer (pH 7.4) for NADPH, was run in parallel and terminated at 40 minutes. Samples were analyzed by HPLC-MS/MS, and peak areas were recorded. The percentage of test compound remaining relative to the zero-time point (%Remained) was plotted against incubation time. The rate of metabolism (k) was determined from the slope of the linear regression of log %Remained versus time, and the in vitro half-life (T_1/2_) was calculated as -0.693/k.

**Hepatocyte Stability Assay:** The PB200 stock solution was diluted to 500 μM with a 1:1 mixture of methanol and H_2_O. Stock solutions (10 mM in 100% DMSO) of positive controls testosterone and 7-ethoxycoumarin were similarly diluted to 500 μM or 100 μM. For all diluted solutions, the final DMSO and methanol concentrations were ≤ 0.1%. Incubations were conducted in duplicate in 96-well plates. Each well contained 50 μL of Williams E medium with 1x Glutamax, 1 million hepatocytes/mL, and either test compound (5 μM) or a positive control (5 μM or 1 μM). Reactions were terminated at 0, 15, 30, 60, and 120 minutes by adding 200 μL of ice-cold acetonitrile with an internal standard. The plates were centrifuged (4000 rpm, 20 min), and 50 μL from each sample was transferred to a daughter plate, to which 200 μL of ddH_2_O was added per well. Samples were then analyzed using LC/MS/MS.

**Mice brain/plasma PK studies of** **PB200^3, 4^**

*In vivo* mice brain and plasma PK studies were conducted by HD Biosciences (China) Co., Ltd. Male C57BL/6 mice (6–8 weeks old; n = 3 per time point) were acclimated for 3 days prior to experimentation and confirmed to be healthy. PB200 was formulated in 10% DMSO/10% Tween 80/80% saline at a final concentration of 1 mg/mL. Each mouse received a single injection (*i.v*.) of approximately 25 μL of formulation. At designated time points (0.5, 1, and 4 hours post-dose), ~80 μL of blood was collected from the submandibular vein into EDTA-2K-coated tubes, mixed, and immediately placed on ice. Samples were centrifuged at 4000 rpm, 4 °C for 10 minutes to obtain plasma (~30 μL), which was stored at –80 °C for later analysis. Immediately following blood collection, brains were rapidly harvested, rinsed with cold 0.9% saline, blotted dry, weighed, and stored at –70 °C. Within 1 hour, brains were homogenized in cold PBS (pH 7.4) on ice and aliquots stored at –70 °C until analysis.

For plasma analysis, 20 μL of plasma was mixed with 80 μL acetonitrile containing internal standard, vortexed, and centrifuged at 14,000 rpm for 5 minutes. Supernatant (80 μL) was diluted with 320 μL H_2_O, and the mixture subjected to LC-MS/MS (Kinetex C18 column, 2.6 μm, 2.1 × 50 mm, 100 Å) for quantification. Brain homogenates were processed similarly using acetonitrile precipitation and analyzed by LC-MS/MS. PB200 concentrations in plasma (ng/mL) and brain (ng/g) were determined, and plasma-to-brain ratios were calculated.


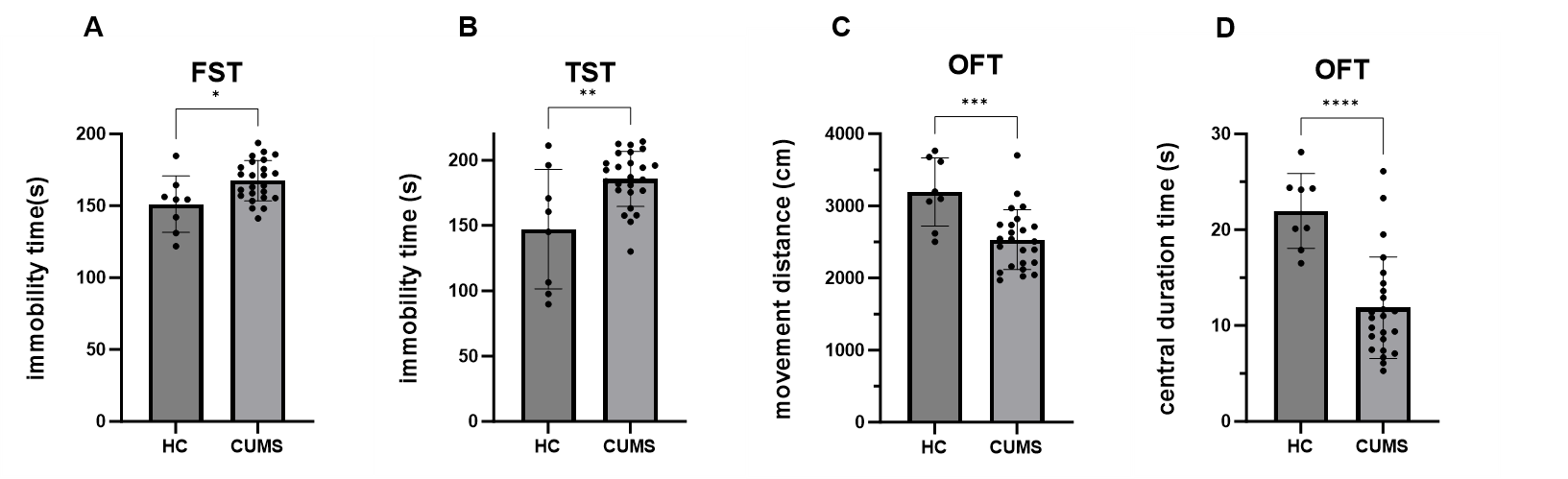


**Figure S4. Behavioral experimental results of the CUMS-Induced MDD Model.** (A) CUMS mice showed a significant increase in immobility time in the forced swim test (FST) and (B) the tail suspension test (TST). (C) In the open field test (OFT), CUMS mice displayed reduced locomotor activity, evidenced by a decrease in the total distance moved, and (D) increased anxiety-like behavior, shown by a significant reduction in time spent in the central area of the arena. Data are presented as mean ± SEM, n = 8 for HC, n = 24 for CUMS, p < 0.05, p < 0.01, *p < 0.001, **p < 0.0001 by two-tailed unpaired Student’s t-test.

# **NMR Spectrum of synthetic compounds**


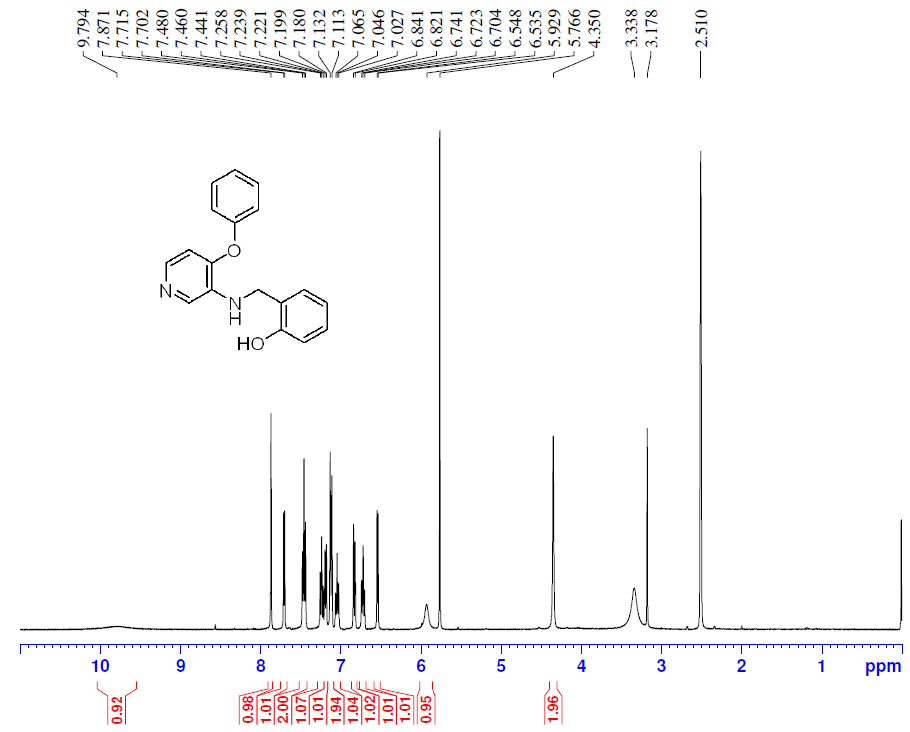


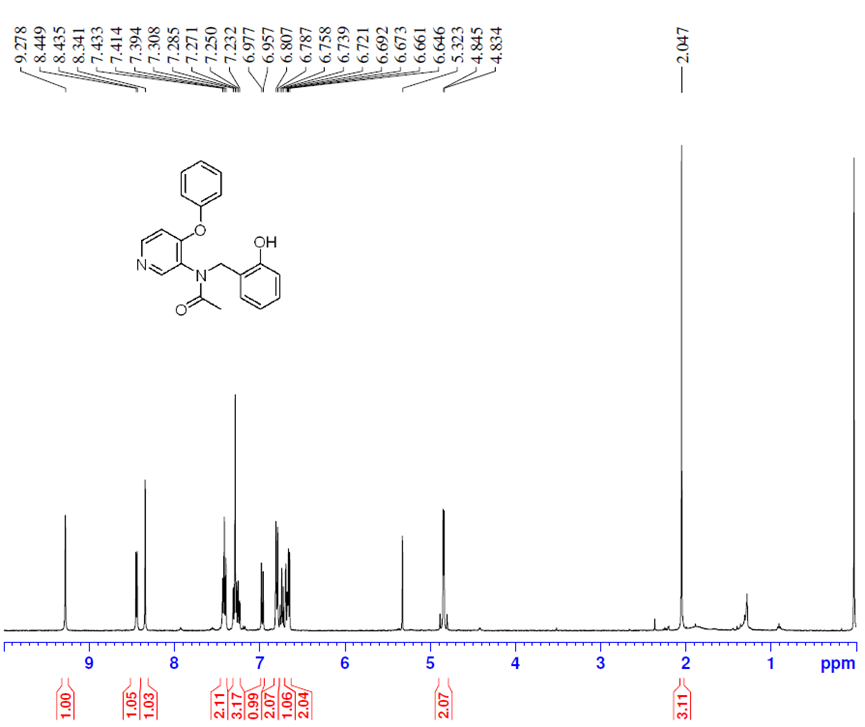


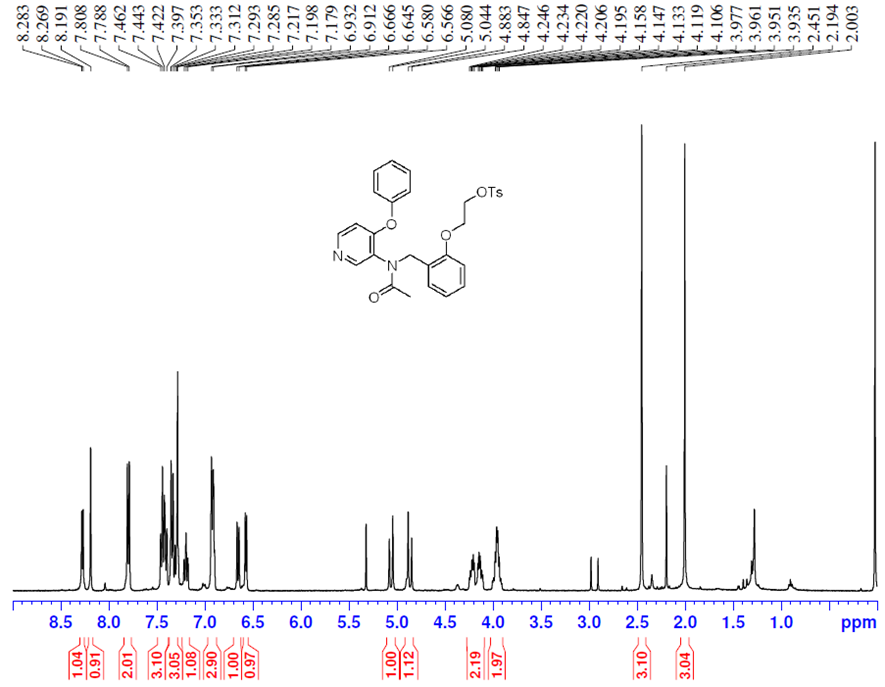


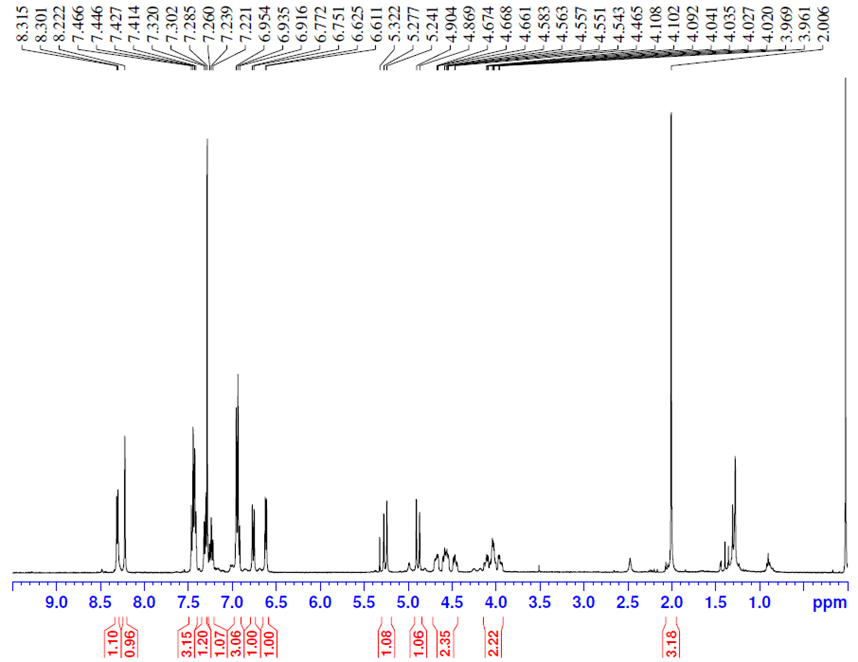


^1^**H-NMR Spectrum (400 MHz, DMSO-*d_6_*) of Compound 3a**


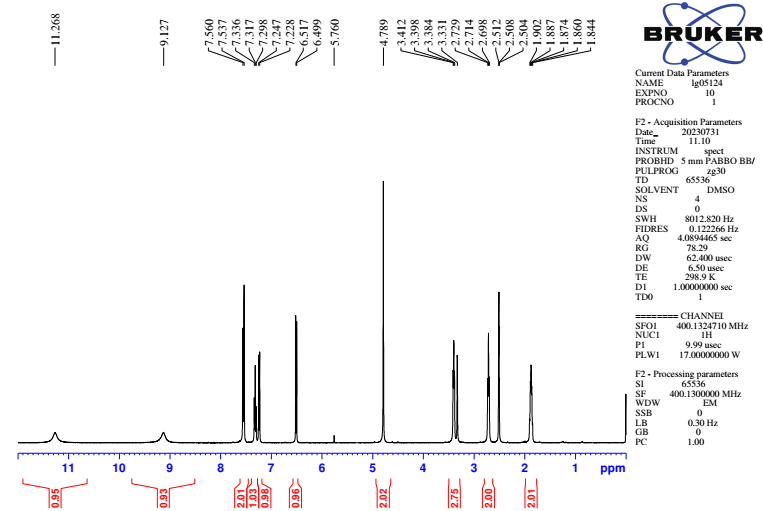


^13^**C-NMR Spectrum (100 MHz, DMSO-*d_6_*) of Compound 3a**


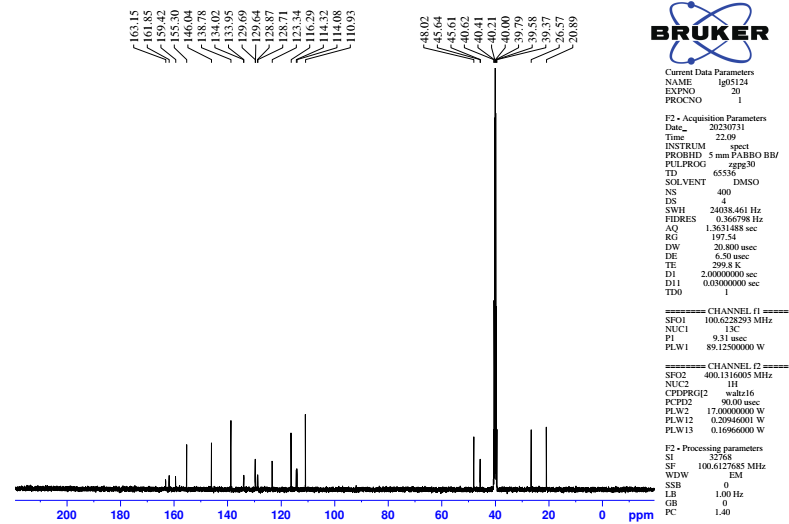


^1^**H-NMR Spectrum (400 MHz, DMSO-*d_6_*) of Compound 3b**


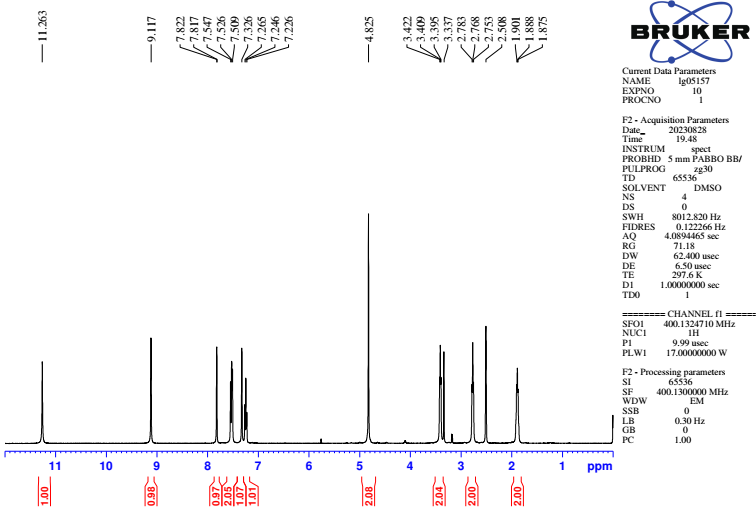


^13^**C-NMR Spectrum (100 MHz, DMSO-*d_6_*) of Compound 3b**


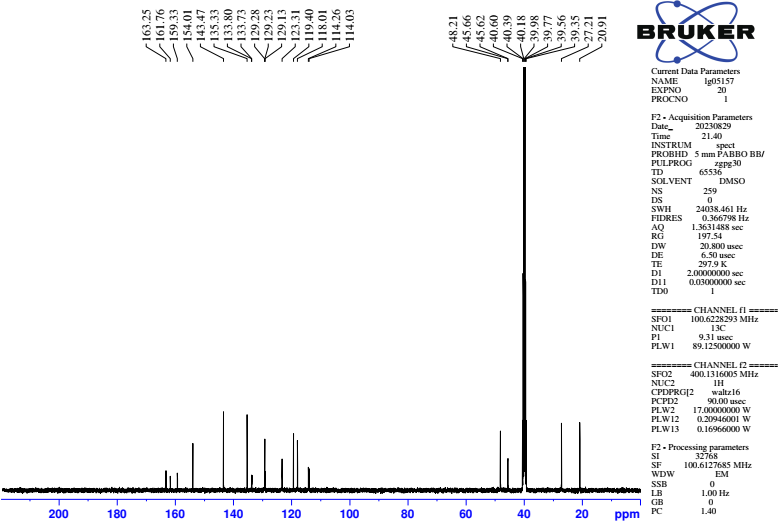


^1^**H-NMR Spectrum (400 MHz, DMSO-*d_6_*) of Compound 3c**


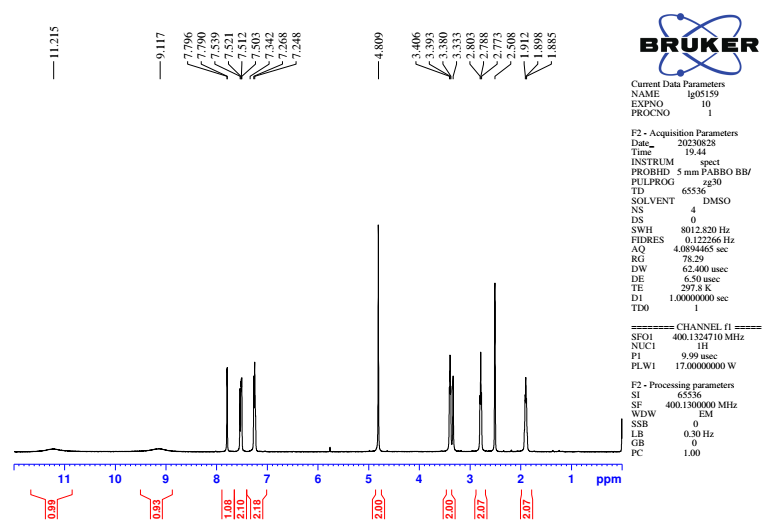


^13^**C-NMR Spectrum (100 MHz, DMSO-*d_6_*) of Compound 3c**


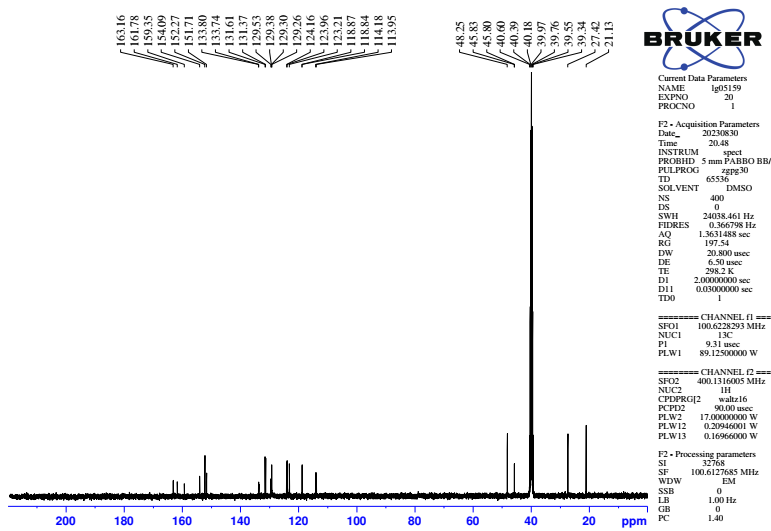


^1^**H-NMR Spectrum (400 MHz, DMSO-*d_6_*) of Compound 3d**


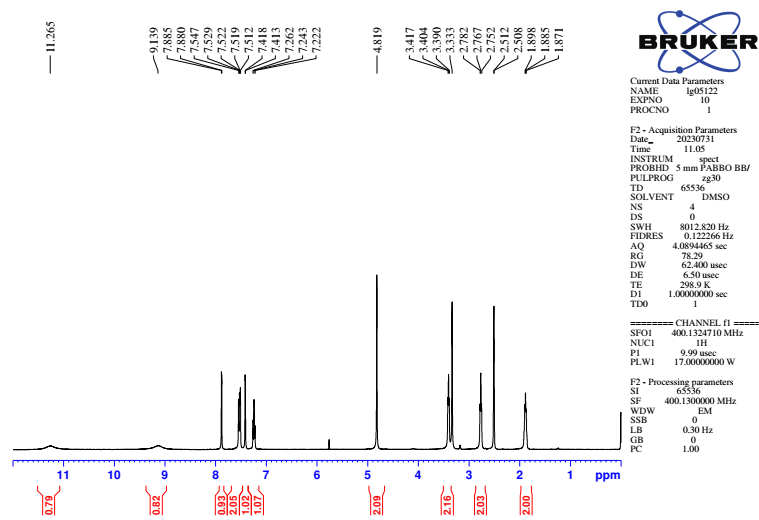
^13^**C-NMR Spectrum (100 MHz, DMSO-*d_6_*) of Compound 3d**


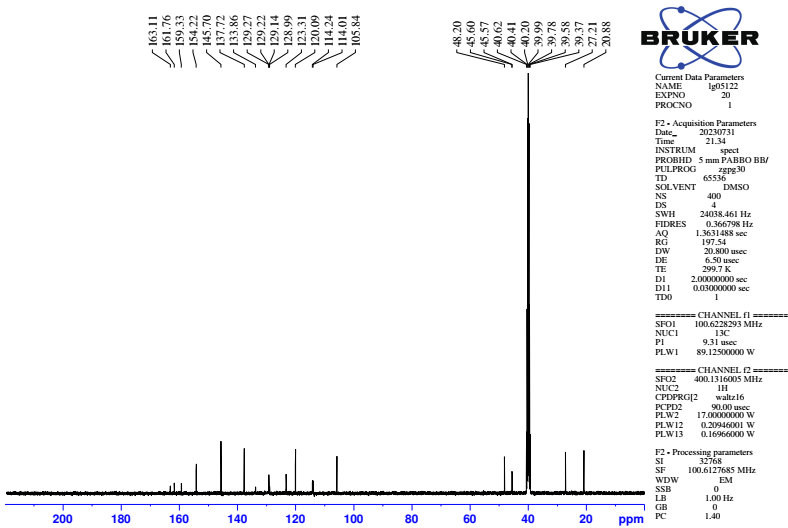


^1^**H-NMR Spectrum (400 MHz, DMSO-*d_6_*) of Compound 3e**


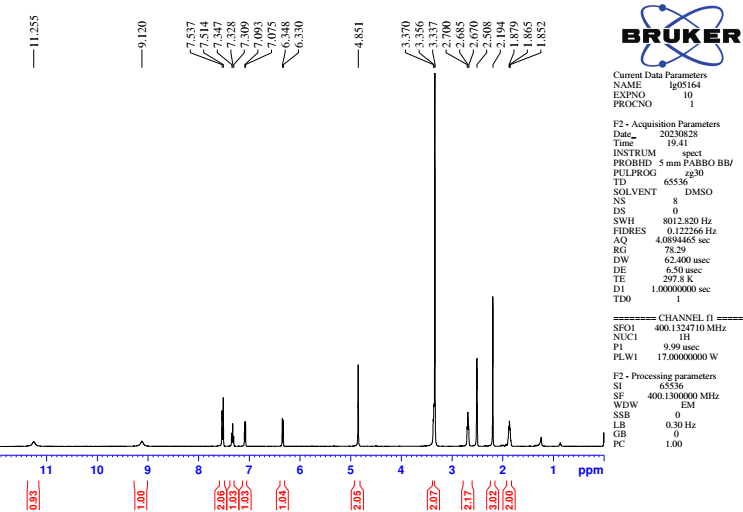


^13^**C-NMR Spectrum (100 MHz, DMSO-*d_6_*) of Compound 3e**


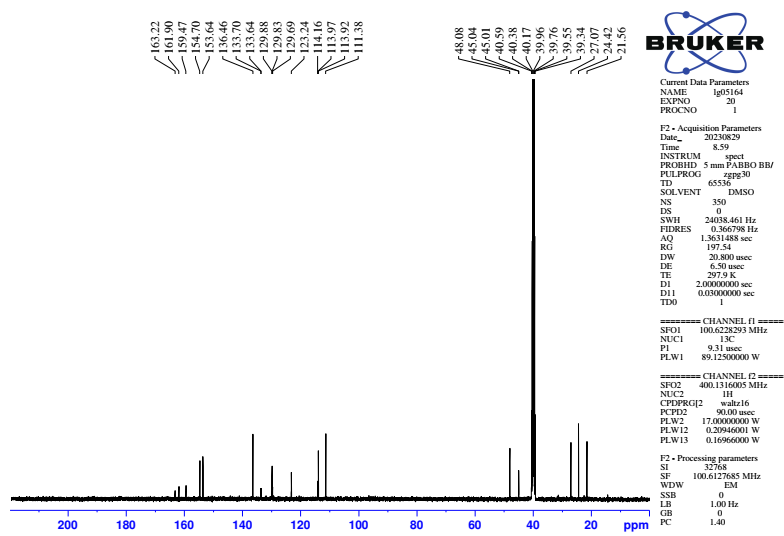


^1^**H-NMR Spectrum (400 MHz, DMSO-*d_6_*) of Compound 3f**


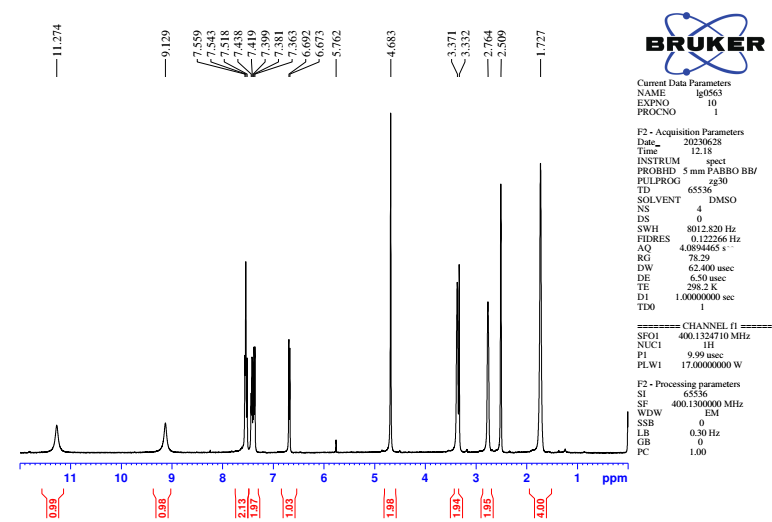
^13^**C-NMR Spectrum (100 MHz, DMSO-*d_6_*) of Compound 3f**


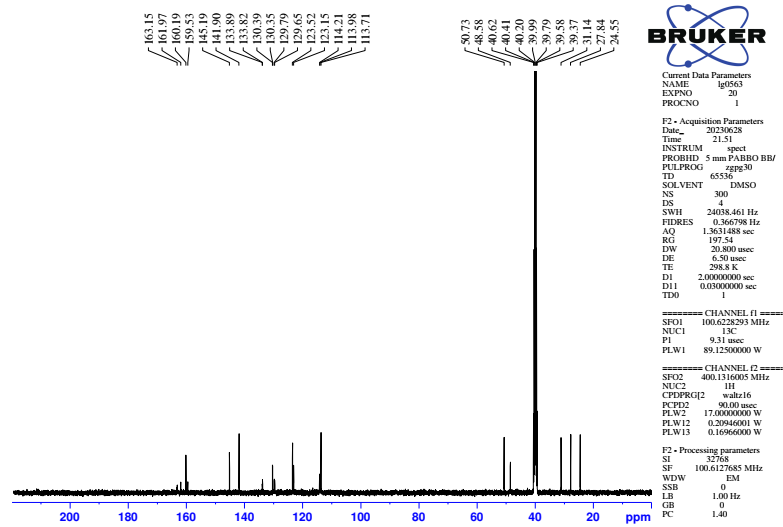


^1^**H-NMR Spectrum (400 MHz, DMSO-*d_6_*) of Compound 3g**


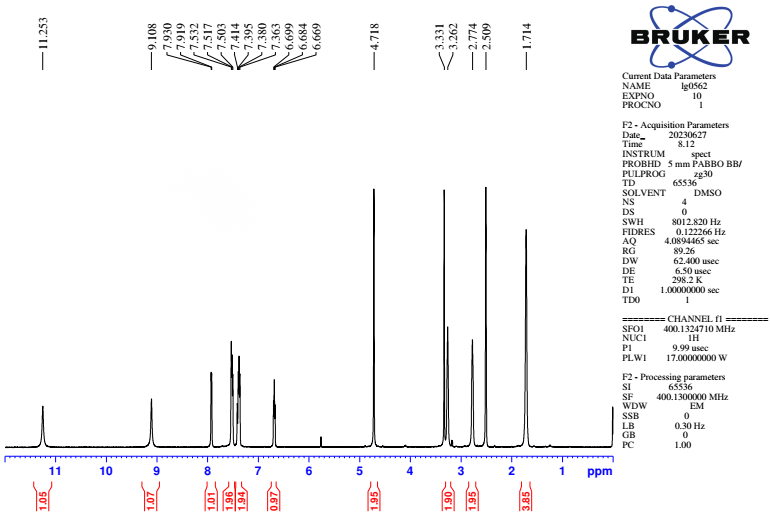


^13^**C-NMR Spectrum (100 MHz, DMSO-*d_6_*) of Compound 3g**


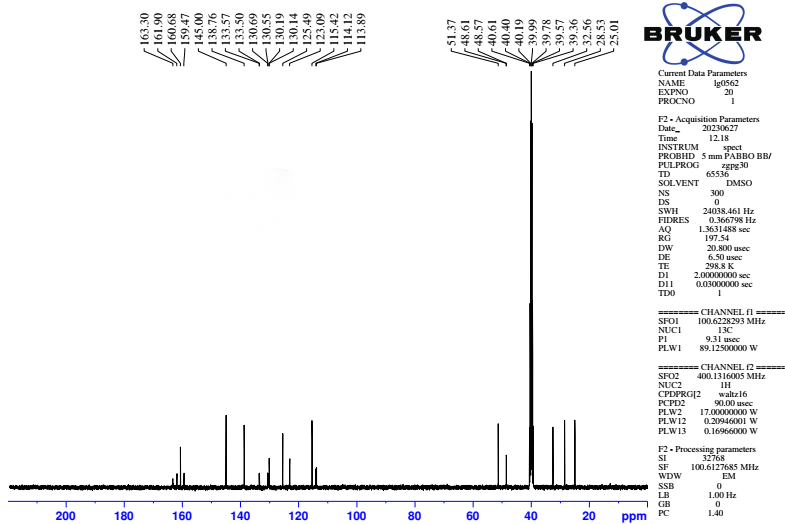


^1^**H-NMR Spectrum (400 MHz, DMSO-*d_6_*) of Compound 3h**


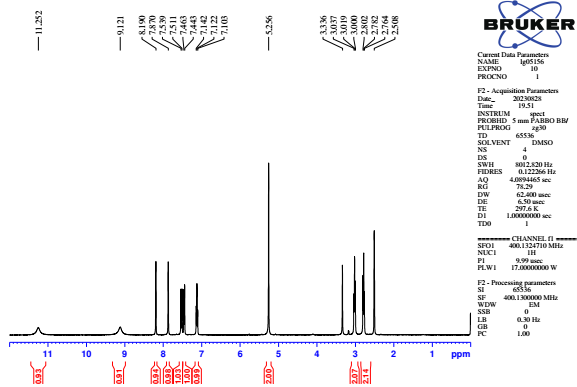


^13^**C-NMR Spectrum (100 MHz, DMSO-*d_6_*) of Compound 3h**


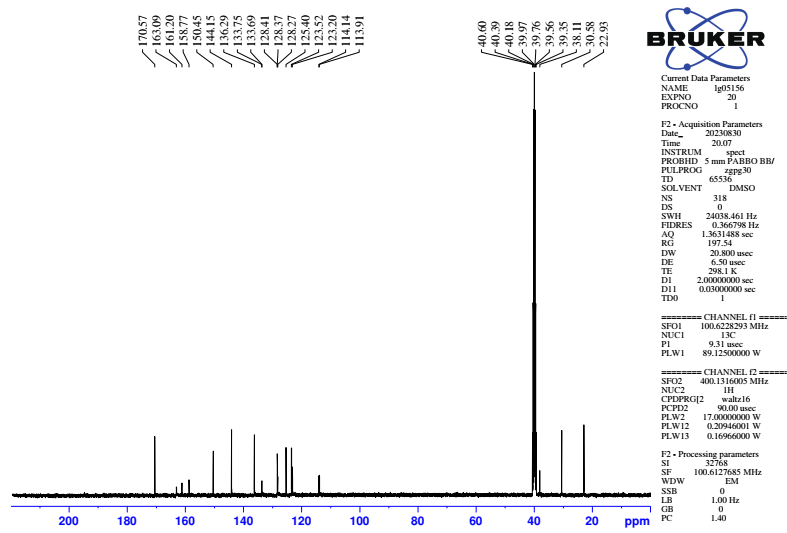


# **References**

1. R.S. Obach, J.G. Baxter, T.E. Liston, B.M. Silber, B.C. Jones, F. MacIntyre, D.J. Rance, P. Wastall. The prediction of human pharmacokinetic parameters from preclinical and in vitro metabolism data. *J Pharmacol Exp Ther* 1997**,** **283** (1): 46-58.

2. L. Di, E.H. Kerns, Y. Hong, H. Chen. Development and application of high throughput plasma stability assay for drug discovery. *Int J Pharm* 2005**,** **297** (1-2): 110-9.

3. C.D. Bevan, R.S. Lloyd. A high-throughput screening method for the determination of aqueous drug solubility using laser nephelometry in microtiter plates. *Anal Chem* 2000**,** **72** (8): 1781-7.

4. K.P. Bateman, J. Castro-Perez, M. Wrona, J.P. Shockcor, K. Yu, R. Oballa, D.A. Nicoll-Griffith. MSE with mass defect filtering for in vitro and in vivo metabolite identification. *Rapid Commun Mass Spectrom* 2007**,** **21** (9): 1485-96.
